# *KAT6A*-Mutant iPSC-Derived Cortical Neurons Display Precocious Maturation, Dysregulated Electrophysiology, and Altered Morphology

**DOI:** 10.7150/ijbs.134210

**Published:** 2026-09-24

**Authors:** Naama Wiesel-Motiuk, Ashwani Choudhary, Yara Hussein, Utkarsh Tripathi, Wote Amelo Rike, Aviram Shemen, Ritu Nayak, Souvik Chakraborty, Omveer Sharma, Tatiana Rabinski, Michal Stark, Karin Weiss, Gad D. Vatine, Shani Stern, Yehuda G. Assaraf

**Affiliations:** 1 The Fred Wyszkowski Cancer Research Laboratory, Faculty of Biology, Technion-Israel Institute of Technology, Haifa 3200003, Israel.; 2 Sagol Department of Neurobiology, Faculty of Natural Sciences, University of Haifa, Haifa 3498838, Israel.; 3 The Department of Physiology and Cell Biology, Faculty of Health Sciences, Ben-Gurion University of the Negev, Beer Sheva 8410501, Israel.; 4 The Regenerative Medicine and Stem Cell (RMSC) Research Center. Ben-Gurion University of the Negev, Beer Sheva 8410501, Israel.; 5 The Zelman Center of Neuroscience, Ben-Gurion University of the Negev, Beer Sheva 8410501, Israel.; 6 Genetic Institute, Rambam Health Care Campus, Haifa 3109601, Israel.; 7 Ruth and Bruce Rappaport Faculty of Medicine, Technion - Israel Institute of Technology, Haifa 3109601, Israel.

**Keywords:** KAT6A, KAT6B, lysine acetyltransferase, neurodevelopmental syndromes, autism spectrum disorder, molecular neuroscience

## Abstract

KAT6A syndrome is a complex neurodevelopmental disorder caused by heterozygous pathogenic *KAT6A* variants inflicting variable degrees of developmental delay and intellectual disability via a mechanism that remains largely uncharacterized. To further our understanding, we differentiated KAT6A syndrome patient-derived induced pluripotent stem cells into neural progenitor cells and cortical neurons. KAT6A syndrome patient-derived cells exhibit an altered transcriptome, with many of the dysregulated genes associated with autism spectrum disorder. At an early stage of differentiation, *KAT6A*-mutant cortical neurons show many elevated neurodevelopment-specific genes, including key transcription factors. Detailed electrophysiological measurements uncover that *KAT6A*-mutant cortical neurons display a hyperexcitability phenotype at an early stage of differentiation, followed by hypoexcitability at a later stage, compared with control neurons. These electrophysiological alterations were accompanied by decreased sodium and potassium currents. Remarkably, early *KAT6A*-mutant neurons exhibit a markedly lower percentage of GABA-positive neurons and increased spontaneous calcium activity as compared with control neurons. Moreover, multiple morphometric alterations in early-stage *KAT6A*-mutant cortical neurons indicate premature maturation, including increased neurite outgrowth, arbor complexity and soma size. Consistently, *KAT6A* knockout SH-SY5Y cells exhibit impaired differentiation capability and dysregulated gene expression. These novel findings establish the deleterious impact of pathogenic *KAT6A* variants on cortical neuron differentiation and electrophysiological function.

## Introduction

KAT6A (formerly known as MOZ and MYST3) is a lysine acetyltransferase which belongs to the MYST family of acetyltransferases, along with its paralog KAT6B (formerly known as MORF and MYST4) 1,2. Lysine acetyltransferases, including KAT6A and KAT6B, catalyze the acylation of lysine residues on histones and non-histone proteins 3. By post-translationally modifying lysine residues, KAT6 acetyltransferases play an essential role in the regulation of many important cellular processes, including transcription, cell cycle progression, cell proliferation and differentiation, various developmental processes as well as the maintenance of hematopoietic and neuronal stem cells 4,5.

Proper histone modifications are essential for precise regulation of gene expression 6. This tight regulation over the level and spatiotemporal pattern of gene expression is critical for accurate cell proliferation, differentiation and development, with particular importance for neural development. Hence, dysregulation of histone-modifying enzymes and histone modifications are consistently associated with neurodevelopmental disorders 2,4,6,7. Specifically, pathogenic variants in genes encoding acetyltransferases, such as *CREBBP* (*KAT3A*), *EP300* (*KAT3B*), *KAT5*, *KAT6A*, *KAT6B* and *KAT8*, were linked to various neurodevelopmental disorders (OMIM# 180849, # 613684, # 618332, # 618333, #619103, # 616268, #606170, #603736, #618974) 6. The majority of these acetyltransferases have been shown to regulate the proliferation and differentiation of neural stem/progenitor cells and/or impact neurogenesis as well as cerebral development 8. The molecular and cellular etiology of the clinical phenotypes associated with these syndromes remains to be elucidated 6.

*KAT6A* is located on chromosome 8 (8p11.21) and was first discovered as a target of recurrent chromosomal translocations, resulting in acute myeloid leukemia (AML) 9,10. Since then, pathogenic variants in *KAT6A* and its aberrant expression have been found in an assortment of cancers 4,5. Furthermore, heterozygous pathogenic variants in *KAT6A* were found to be the cause of a neurodevelopmental disorder, now called KAT6A syndrome, also known as the Arboleda-Tham syndrome, OMIM#616268 11-13. Most of the pathogenic variants reported so far in KAT6A syndrome patients are *de novo* loss-of-function (LoF) truncating variants, while some missense variants were also identified 14,15. KAT6A syndrome is primarily characterized by developmental delay and variable degrees of intellectual disability (ID). While most patients display craniofacial abnormalities, speech delay, and hypotonia, other patients exhibit additional neurological and developmental symptoms such as autistic behavior, epilepsy, sleep disturbances, visual defects, cardiac anomalies, as well as gastrointestinal and feeding problems 4,14,16,17. Magnetic Resonance Imaging (MRI) scans of the brain did not identify structural anomalies in most KAT6A syndrome patients 14. However, some patients were reported to have microcephaly, and rare structural anomalies that were reported in several patients include craniosynostosis, pituitary malformation, thin corpus callosum as well as large cisterna magna 14. Studies aiming to characterize the cognitive and neurobehavioral profile of individuals with pathogenic *KAT6A* variants found that most of the patients display an impairment in self-care skills, socialization as well as impairment of both receptive and expressive language skills 15,17,18. Non-verbal cognition is also impaired in KAT6A syndrome patients 18. Although individuals with KAT6A syndrome experience significant cognitive difficulties, they exhibit low rates of behavioral problems 15,18. While they are able to regulate their emotions and show social drive, they exhibit autism-related features, such as inflexible behavior and impaired adaptive skills 18.

Knockout (KO) of *Kat6a* in mice resulted in the disruption of early development, leading to embryonic lethality 19-21. Studies in mice and zebrafish revealed that KAT6A modulates the expression of multiple homeodomain transcription factor (TF) genes that are crucial for regulation of development. Among these genes are the HOX family genes, which regulate body segment identity 19,22,23, and DLX genes, which control craniofacial development 24.

The molecular and cellular mechanisms underlying the manifestation of KAT6A syndrome, and their specific effect on neurodevelopment, remain largely unknown. A recent study in mice revealed that KAT6A deficiency in the hippocampus led to impaired synaptic structure and plasticity in CA3 (but not in CA1) hippocampal neurons, consequently attenuating memory formation 25. This synaptic impairment was found to be the consequence of downregulated RSPO2-mediated Wnt signaling. Additionally, Eccles *et al.,* reported that *Kat6a* haploinsufficient mice exhibit hyperactivity as well as cognitive deficits 26. They further observed a decrease in global histone H3 lysine 23 acetylation (H3K23ac) in the brain and peripheral white blood cells of *Kat6a*^+/-^ mice and consistently in human HEK293T cells carrying human pathogenic *KAT6A* variants. These mice further displayed an altered transcriptome in the dorsal telencephalon at E12.5 and in E16.5 cortical neurons.

In the current study, we used cortical neurons differentiated from iPSCs derived from a KAT6A syndrome patient and two healthy donors, in order to characterize the impact of a pathogenic *KAT6A* variant on the electrophysiological characteristics of cortical neurons. We also examined gene expression profiles in neural progenitor cells (NPCs) and cortical neurons, differentiated from these iPSCs, using RNA sequencing. We show that young *KAT6A*-mutant neurons exhibit accelerated differentiation at an early differentiation stage, involving morphometric growth alterations and hyperexcitability, while *KAT6A*-mutant neurons that were differentiated for a longer period of time became hypoexcitable. We have previously reported similar phenotypes of early maturation and hyperexcitability, where later the neurons degrade and become hypoexcitable with less synaptic connections in other neurodevelopmental and autism spectrum disorder (ASD) related variants and idiopathic patients 27-30. Gene expression profiles of NPCs as well as cortical neurons, at two time points along their differentiation, revealed many dysregulated genes in *KAT6A*-mutant cells and neurons. Moreover, we show that *KAT6A* KO impairs the differentiation capacity of SH-SY5Y cells, a *bona fide* human neuronal differentiation and function model, treated with retinoic acid (RA) and brain-derived neurotrophic factor (BDNF). We present a comprehensive investigation and characterization of the pathophysiological changes in *KAT6A*-mutant NPCs and cortical neurons. These findings enhance our ability to identify therapeutic targets for KAT6A syndrome towards the development of possible therapeutic interventions.

## Materials and Methods

### Reprogramming of dermal fibroblasts into iPSCs

Skin biopsies were obtained from a KAT6A syndrome patient and her healthy mother following informed consent at CHOP (Children's Hospital of Philadelphia, PA, USA). The study was approved by the Technion's Institutional Review Board (IRB 228-2025). Two independent clones of iPSCs were generated from dermal fibroblasts derived from a 7-year-old female patient carrying a *de novo* heterozygous frameshift variant (c.4025delA) in *KAT6A*, identified by clinical exome sequencing (Fig. S1). The patient exhibited phenotypes of developmental delay affecting her motor and language skills. Her clinical characterization included speech delay, hypotonia in trunk and hands, motor delay and disjointed gait, atrial septal defect; she experienced intermittent strabismus, feeding difficulties and sleep disturbances. She didn't have seizures and MRI was normal.

Control iPSCs were generated from dermal fibroblasts derived from the patient's mother (Fig. S1), and a non-related female control iPSC line previously described 31. All new iPSC lines were generated and characterized as previously described 31. In brief, a total of 0.5-1x10^6^ cells were collected with TrypLE (Gibco, Thermo Fisher Scientific, Waltham, MA, USA) and electroporated with non-integrating episomal vectors using a Neon transfection system (Invitrogen, Carlsbad, CA, USA). Cells were cultured in DMEM supplemented with 15% FBS, 5 ng/ml basic fibroblast growth factor (bFGF, peprotech, NJ, USA), and 5 µM ROCK inhibitor on mouse embryonic fibroblast (MEF)-coated plates (Sigma-Aldrich, St. Louis, MO, USA). After 2 days, Nutristem medium (Biological Industries, Beit HaEmek, Israel) supplemented with 5 ng/ml bFGF was utilized with medium refreshment every other day. Colonies were selected and transferred to MEF-coated plates with Nutristem medium containing 5 ng/ml bFGF. Three colonies were selected and manually transferred to Matrigel (Corning)-coated plates with Nutristem medium, which was replenished daily.

### Characterization of iPSCs

iPSCs were characterized as previously described 31. In brief, the retention of the pathogenic *KAT6A* variant in the iPSC lines was verified using Sanger sequencing of genomic DNA (Fig. S1A). Genomic DNA was isolated using TriReagent according to the manufacturer's protocol (T9424, Sigma-Aldrich). PCR was performed using Hy-Taq Ready Mix (#EZ3007, Hylabs, Rehovot, Israel); primers are listed in Supplementary Table S5. G-band karyotype analysis confirmed that the iPSC lines showed no chromosomal abnormalities (Fig. S1B). Pluripotency was confirmed using immunofluorescence as well as flow cytometry analysis of OCT3/4, SSEA4, and TRA-1-60 markers (Fig. S1D, E). iPSCs gave rise to embryoid bodies (EBs) (Fig. S1F), and spontaneous differentiation into the three germ layers was validated by immunofluorescence for three germ layer markers, Neurofilament (NF66), α-SMA, and α-fetoprotein (Fig. S1G). All lines were screened for mycoplasma contamination using the Hy mycoplasma PCR kit (Hylabs).

### iPSC culturing and generation of NPCs

iPSCs were cultured and maintained using mTesR Plus medium (#100-0276, StemCell Technologies, Canada). Cortical neurons were generated using a previously described protocol 32. In brief, iPSCs were grown to approximately 80% confluency, then dissociated using 1 ml of Dispase (#07923, StemCell Technologies) and plated onto low-adherence plates (#351007, Falcon) in mTeSR medium supplemented with 10 µM ROCK inhibitor (#72307, StemCell Technologies), allowing for the formation of EBs. The next day, the medium was replaced with mTesR Plus medium without ROCK inhibitor. In the following 10 days, the cells were fed with EB medium: DMEM/F12 supplemented with 2 mM Glutamax (#35050038, Gibco), 2% B27 supplement (#17504044, Gibco), 1% N2 supplement (#17502048, Gibco), and 0.1 μM LDN193189 Hydrochloride (#1066208, Biogems, Westlake Village, CA, USA). The EBs were plated onto poly-L-ornithine/laminin (#P3655, Sigma-Aldrich, #3400-010-03, R&D Systems, Minneapolis, MN, USA) coated six-well dishes and fed with EB medium containing 1 µg/ml laminin (#23-017-015, Gibco) for the following 7 days to allow for the formation of neural rosettes. The rosettes were selected based on their morphology and were manually picked, dissociated with Accutase (#07920, StemCell Technologies), and plated onto poly-L-ornithine/laminin-coated dishes in NPC medium: DMEM/F12 supplemented with 2 mM Glutamax, 2% B27 supplement, 1% N2 supplement, 1 µg/ml laminin, and 20 ng/ml bFGF (# PHG0264, Gibco). A complete medium change was performed every other day until full confluency was achieved.

### Differentiation of NPCs into cortical neurons

NPCs were differentiated into cortical neurons using a differentiation medium containing: DMEM/F12, 2 mM Glutamax, 2% B27 supplement, 1% N2, 0.2 nM L-Ascorbic Acid (#5088177, Biogems), 500 µg/ml dibutyryl-cAMP (#A15914-5, Adooq, Irvine, CA, USA), 1 µg/ml laminin, 20 ng/ml BDNF (#AF-450-02, Peprotech), and 20 ng/ml GDNF (# 450-10, Peprotech) for 10 days. Between days 11 and 14, the cells were dissociated again and seeded on coverslips and then fed with Brainphys-based medium (#05790, STEMCELL Technologies) supplemented with 2 mM Glutamax, 2% B27 supplement, 1% N2, 0.2 nM L-Ascorbic Acid, 500 µg/ml dibutyryl-cAMP, 1 µg/ml laminin, 20 ng/ml BDNF, and 20 ng/ml GDNF.

### Electrophysiology

Whole-cell patch-clamp recordings were performed on neurons derived from two clones of *KAT6A*-mutant cortical neurons, and neurons derived from two healthy control individuals, 4-5 weeks after the start of the differentiation for the 1^st^ tp and 9-10 weeks post-differentiation for the 2^nd^ tp. Culture coverslips were placed inside a recording chamber filled with HEPES-based artificial cerebrospinal fluid (ACSF) containing: 10 mM HEPES (#H0887, Sigma-Aldrich), 139 mM NaCl (#S9888, Sigma-Aldrich), 4 mM KCl (#P3911, Sigma-Aldrich,), 2 mM CaCl_2_ (#223506, Sigma-Aldrich), 10 mM D-glucose (#G7021, Sigma-Aldrich), and 1 mM MgCl_2_ (#7791-18-6, Merck), at pH 7.4 and osmolarity was adjusted to 305-310 mOsm/L. Fire-polished borosilicate glass capillaries (#BF150-75-10, Sutter Instrument, Novato, CA, USA) were pulled (tip resistance of about 9-12 MΩ) and filled with an internal solution containing: 130 mM K-gluconate (#G4500, Sigma-Aldrich), 6 mM KCl, 4 mM NaCl, 10 mM Na-HEPES (#H3784, Sigma-Aldrich), 0.2 mM EGTA (#03777, Sigma-Aldrich), 0.3 mM GTP (#51120, Sigma-Aldrich), 2 mM Mg-ATP (#A9187, Sigma-Aldrich), 0.2 mM cAMP, and 10 mM D-glucose. All measurements were performed at room temperature using a patch clamp amplifier (MultiClamp 700B, Molecular Devices, San Jose, CA, USA), connected to a digitizer (Axon Digidata 1550B, Molecular Devices, San Jose, CA, USA), and controlled by MultiClamp 700B Commander and pCLAMP 11 software. Pipette offset was corrected before seal formation, pipette capacitance was neutralized, and automated bridge-balance compensation was applied, following establishment of the whole-cell configuration prior to current-clamp recordings. Data were acquired at a sampling rate of 20 kHz.

### Analysis of electrophysiological recordings

The acquired data were analyzed using previously described methods 33 and custom-written MATLAB scripts. Briefly:

#### (a) Synaptic currents analysis

Neurons were held in a voltage clamp mode at -60 mV and spontaneous excitatory postsynaptic current (sEPSC) event rate and amplitudes for each active cell were calculated. The frequency of events for each cell (sEPSC rate) was calculated by dividing the number of events by the duration of the recording (including non-active cells, which were assigned an event rate of 0 Hz).

#### (b) Sodium, fast and slow potassium currents

Neurons were held in voltage clamp mode at -60 mV, and voltage steps of 400 ms were applied in the range of -100 to 90 mV. The cells' capacitance typically normalizes currents. The sodium current was computed by subtracting the sodium current after stabilization from the lowest value of the inward sodium current. The fast potassium currents were measured by the maximum outward currents that appeared within a few milliseconds after a depolarization step. The slow potassium currents were measured at the end of the 400 ms depolarization step. Input conductance was calculated by measuring the steady-state current response to small voltage steps around the resting membrane potential. Specifically, the mean current was extracted at holding potentials of -70 mV and -60 mV, and the difference in the current between these two steps was divided by the difference in voltage.

#### (c) Evoked action potentials (APs)

Holding: Neurons were held at -60 mV in a current-clamp mode by adjusting the holding current.

Stimulation: Current was injected in 3 pA steps for 400 ms each, starting 12 pA below the steady-hold current.

Counting: The total count of evoked action potential was the total number of action potentials that were counted in the 38 depolarization steps.

#### (d) Spontaneous APs

**Holding:** Neurons were held at -45 mV in a current-clamp mode by adjusting the holding current.

**Recording:** Spontaneous spikes were counted during a 51-second window.

**Counting:** The latter represents the total number of action potentials counted.

### Calcium imaging and analysis

Calcium imaging experiments were performed on cortical neurons derived from two *KAT6A*-mutant clones and from two healthy donor lines at the same two time points indicated for the electrophysiology recordings. To monitor spontaneous calcium activity, cortical neuron cultures were incubated for 1 hour in culture medium containing 2 μM Fluo-5 AM, a cell permeant green fluorescent calcium probe (AB241083, abcam). Following incubation, the coverslips were washed once with pre-warmed ACSF for 5 minutes and then transferred to a recording chamber containing fresh pre-warmed ACSF. Calcium transients were recorded using a Leica THUNDER imager fluorescence microscope at a sampling rate of 10 Hz. For each coverslip, 10-12 independent recordings were acquired, each consisting of a 3-minute recording (i.e., 1,800 frames). Fluorescence intensity traces were extracted for individual neurons from each recording and analyzed using custom-written MATLAB scripts (R2026a, MathWorks). To reduce high-frequency noise, fluorescence traces were smoothed before analysis. Calcium transients were identified using an automated peak-detection algorithm based on peak prominence, peak distance, and peak width. Cells exhibiting at least one detectable calcium transient were classified as active neurons. For each region of interest, fluorescence traces were normalized to the baseline fluorescence and expressed as ΔF/F_0_ according to the equation ΔF/F_0_ = (F - F_0_)/F_0_, where *F* is the fluorescence intensity at each acquired frame and F_0_ is the baseline fluorescence intensity.

For each recording, the following parameters were quantified: the total number of calcium transients, mean event frequency, average peak width, average peak prominence (peak amplitude relative to the surrounding baseline), and the percentage of active neurons. Furthermore, the kinetics of calcium transients were assessed by measuring the rise time (i.e., time from the estimated onset of the event to the peak) and the rise slope (rate of fluorescence increase during the rising phase).

### RNA extraction, sequencing and analysis

Total RNA from two to three million cells per sample (NPCs and cortical neurons at 1^st^ tp and 2^nd^ tp of differentiation, derived from two patient clones with the pathogenic *KAT6A* variant, and two healthy control lines) was extracted using TRIzol (#15596026, Invitrogen). The isolated aqueous phase was mixed with 100% ethanol at 1:1 ratio and subsequently purified using the Zymo RNA clean & concentrator kit as per the manufacturer's instructions (#R1017, Zymo Research, Irvine, CA, USA). Two biological replicates were performed for each cell line (Except for the unrelated control neurons at 2^nd^ tp, for which we had one sample).

RNA QC, RNA-seq library preparation and sequencing were conducted by the Technion Genomics Center, Technion- Israel Institute of Technology. Quality and concentration measurements for total RNA were performed using the TapeStation 4200 (Agilent, Santa Clara, CA, USA) with the RNA kit (#5067-5576, Agilent). All samples exhibited high integrity (RIN>9.6). 23 RNA-seq libraries were constructed simultaneously using NEBNext UltraExpress RNA Library Prep Kit for Illumina (#E3330, NEB, Ipswich, MA, USA). 100 ng total RNA was used as starting material. mRNAs pull-down was performed using the NEBNext® Poly(A) mRNA Magnetic Isolation Module (#E7490, NEB). RNA-seq library QC was performed by measuring library concentration using Qubit (Invitrogen) with the Equalbit dsDNA HS Assay Kit (#EQ121, Vazyme, Nanjing, China) and size determination using the TapeStation 4200 (Agilent) with the High Sensitivity D1000 kit (#5067-5584, Agilent). All libraries were mixed into a single tube with equal molarity.

The RNA-seq data were generated on Illumina NextSeq2000, using P3 XLEAP-SBS Reagent Kit (200 cycles) (Read1-100; Index1-8; Index2-8; Read2-100) (#20100989, Illumina, San Diego, CA, USA). To analyze the RNA sequencing data, sequencing reads using a next-generation sequencing (NGS) platform were processed in FASTQ-format files. Sequences were quality-checked using the FASTQC algorithm and then aligned to the hg38 human genome using the STAR aligner version 2.78a. Mapping was carried out using default parameters, filtering non-canonical introns, allowing up to 10 mismatches per read, and only keeping uniquely mapped reads. The expression levels of each gene were quantified by counting the number of reads that aligned to each exon or full-length transcript and normalized by its mean across all samples using HTseq v0.9.1. Differentially expressed genes (DEGs) were determined using the DESeq2 algorithm, and the *p*-value was adjusted for multiple hypotheses with the Benjamini-Hochberg procedure, controlling for the false discovery rate (FDR). Genes with an FDR < 0.05 and |log2 fold change | > 0.5 were included in the analysis. To characterize the biological functions and pathways associated with DEGs, Gene Ontology (GO) enrichment and KEGG pathway analysis were performed at the three time points. To assess the potential relevance of dysregulated genes to ASD, gene lists were compared with known ASD-linked genes from the SFARI Gene database (3 April 2025 update release). All the analyses were performed using a custom-written R scripts (Version 4.4.2).

### Immunofluorescence microscopy analysis

Cells grown on 48-well glass coverslips were fixed in 4% paraformaldehyde for 15 minutes and washed with PBS three times. Before blocking the cells with blocking solution (PBS containing 10% horse serum), permeabilization of the cells was performed in blocking solution containing 0.1-0.2% Triton X-100. Coverslips were incubated with the primary antibodies: chicken anti-MAP2 (ab92434, Abcam, Cambridge, UK, 1:500), rat anti-CTIP2 (ab18465, Abcam, 1:400), rabbit anti-VGLUT1 (ab227805, Abcam, 1:500), and Mouse anti-GABA (ab86186, Abcam, 1:400) in blocking solution overnight at 4°C. The next day, coverslips were washed in PBS, incubated with DAPI (ab228549, Abcam, 1:2500) and the corresponding secondary antibodies: Donkey anti-Chicken IgY (H+L), Alexa Fluor 594 (A78951, Thermo Fisher Scientific, 1:500), Donkey Anti-Rat IgG-488 (ab150153, Abcam 1:300), Donkey Anti-Mouse IgG-555 (ab150110, Abcam 1:300), and Donkey Anti-Rabbit IgG-488 (ab150061, Abcam 1:300) for one hour at room temperature, washed again in PBS, mounted on slides using Fluoromount G (Thermo Fisher Scientific), and dried overnight, protected from light. For quantification, images were acquired at 20X magnification using a Leica THUNDER imager. For CTIP2 counting, the images were processed and counted using ImageJ. For VGLUT1 and GABA counting, fluorescence staining was quantified using a custom-written MATLAB script. Artifacts were excluded to prevent outlier signals. For each image, the fluorescence intensity distribution was evaluated using an intensity histogram, and Otsu thresholding 34 was used to determine a threshold to distinguish positive fluorescence from background, thereby minimizing the contribution of nonspecific background fluorescence. Pixels with intensities above the selected threshold were scored as positive. Similar criteria for artifact exclusion, background discrimination and threshold selection were applied consistently across all experimental groups and images.

Confocal images were captured at 20X and 60X using a Nikon Eclipse Ti2-E Spinning Disk Confocal Microscope (Nikon, Minato City, Tokyo, Japan) equipped with a CSU-W1 Confocal Scanner Unit and dual camera (Yokogawa Electric Corporation, Musashino, Tokyo, Japan), using 405 and 561 nm diode lasers. Confocal images were processed using the IMARIS (v10.0.1) software (Oxford Instruments) and images at 20X are shown following projection of Z-stacks using the maximum intensity projection type.

### Morphometric analysis

MAP2-immunostained neuronal images at the 1^st^ tp acquired at 20X as Z-stacks were processed to generate two-dimensional representations of the MAP2-positive neuronal arbor for subsequent morphometric analysis. Neuronal morphology was quantified using a custom semi-automated MATLAB pipeline (MathWorks 2026a; Image Processing Toolbox). Prior to segmentation, MAP2 images underwent background correction and Gaussian filtering. MAP2-positive structures were then segmented using an intensity threshold determined by Otsu's method and manually optimized when needed. Individual neurons were identified by manually marking the center of each neuronal soma on the MAP2 image. Following this initial manual identification, neuronal reconstruction and morphometric analysis were automatically performed. To separate processes belonging to neighboring neurons within the same MAP2-positive network, all manually identified somas were used as seeds for a geodesic distance-based algorithm 35. Each MAP2-positive pixel was assigned to a single neuron according to the shortest path through the MAP2-positive mask from the corresponding soma seed. For each neuron, the reconstructed neuronal mask was skeletonized, allowing quantitative characterization of neurite extension and branching architecture. Morphometric measurements were derived from the neuronal mask and skeleton and included parameters describing soma morphology, neurite length, arbor extent and density, branching and terminal complexity, as well as soma-to-terminal path geometry. Neurite width was estimated from the local distance transform of the MAP2-positive mask along the neurite skeleton, excluding the soma and an adjacent buffer region.

Branching complexity was further assessed using Sholl analysis 36. Concentric rings were generated from the soma at 5 µm intervals, and intersections between each ring and the reconstructed neuronal skeleton were automatically quantified. Thus, the analysis provided complementary measurements of neuronal size, outgrowth, branching complexity, spatial organization, and neurite morphology.

A detailed description of all morphometric parameters quantified by the analysis pipeline is provided in Supplementary Table S6.

### SH-SY5Y cell culture

SH-SY5Y cells (Kindly provided by Prof. Ayelet Fishman, Technion-Israel Institute of Technology) were maintained in growth medium: DMEM/F12 media (01-170-1A, Sartorius, Beit HaEmek, Israel) supplemented with 10% heat-inactivated fetal bovine serum (FBS, A5256701, Gibco) (heat-inactivated at 56°C for 30 min), 2.5 mM L-Glutamine (03-020-1A, Sartorius), 100 μg/mL penicillin-streptomycin (L0022, Biowest, France), in a 5% CO_2_ humidified incubator at 37°C.

*KAT6A* KO SH-SY5Y clones were generated by transfection using the jetOPTIMUS DNA transfection reagent (Polyplus, France) of two LentiCRISPRv2 (Addgene no. 52961) plasmids that contain two different single-guide RNAs (sgRNAs) targeting *KAT6A* (sg1: GCTATTCGCCCAGGATTATC; sg2: CTCGATGCACTGCCACCGTA), following the manufacturer's instructions. Single-cell clones were isolated after selection with 2 μg/ml puromycin (BML-GR312, Enzo Life Sciences, Inc., Farmingdale, NY, USA). The KO was validated by Sanger sequencing of PCR fragments amplified from clones' cDNA (Fig. S2). PCR was performed using Hy-Taq Ready Mix; primers are listed in Supplementary Table S5. Mutated alleles were separated by ligating the PCR fragments into a T-vector (pGEM-T Easy Vector, Promega, USA) according to the manufacturer's instructions. Plasmids from single clones were extracted using GeneJET Plasmid Miniprep Kit (Thermo Scientific) and sequenced using primers listed in Supplementary Table S5.

### Differentiation of SH-SY5Y

Cells were seeded at a density of ~4.7 x 10^4^ cells/cm^2^ in growth medium on 6-well plates for microscope visualization or 60-mm plates for RNA isolation. On day 1 of the differentiation, the medium was replaced with “differentiation medium” (DMEM/F12 supplemented with 1% FBS, 2.5 mM L-Glutamine, 100 μg/mL penicillin-streptomycin, 10μM RA (Stock solutions were diluted in DMSO) (R2625, Sigma-Aldrich). On day 3, the medium was replenished. On day 6, the medium was replaced with serum-free differentiation medium, supplemented with 50 ng/ml BDNF (#B-250, Alomone Labs, Jerusalem, Israel), for 2 days. Phase-contrast images of undifferentiated and differentiated SH-SY5Y cells were acquired using a BioTek Cytation5 microscope (Agilent) at 20X magnification.

### RNA isolation, cDNA synthesis, and real-time PCR analysis

Total RNA was isolated from SH-SY5Y cells using the TriReagent protocol (#T9424, Sigma-Aldrich). cDNA synthesis was carried out using the High-Capacity cDNA Reverse Transcription Kit (Applied Biosystems-Life Technologies, Grand Island, NY, USA) according to the manufacturer's instructions. Real-time PCR was performed using a QuantStudio1 RT-PCR (Applied Biosystem, Foster City, CA, USA). Quantitative PCR was carried out using 1x PerfeCTa qPCR FastMix, LOW ROX (Quanta Biosciences, Gaithersburg, MD, USA). Expression levels were normalized using the *EMC7* gene as an internal control for SH-SY5Y cells, *RPLP0* for NPCs, and *GAPDH* for neurons.

### Statistical analysis

For RNA-seq datasets, statistical analysis was carried out as described in the respective experimental section. MATLAB (version 2023a, 2026a) was used to generate the graphs and statistics for the electrophysiology, calcium imaging experiments, analysis of GABA/VGLUT1 positive cells and morphometric analysis (Data are presented as mean ± SEM). The normality of distributions was assessed using the Kolmogorov-Smirnov test, and the data passed the normality test. Analysis of CTIP2/ MAP2-positive neurons (Data are presented as mean ± SD) were analyzed using Excel. The Shapiro-Wilk normality test was performed to assess the normality of the data distribution on GraphPad Prism (version 8.0), and the data passed the normality test. RT-PCR data (Data are presented as mean ± SD) were analyzed using Excel. Statistical significance of differences was determined using an independent (paired when comparing SH-SY5Y treated cells) two-tailed Student's t-test for two-group comparisons, except for the sodium and potassium currents, which were compared at various potentials using a one-way ANOVA. A *p*-value < 0.05 was considered statistically significant. Significance levels are indicated as follows: *p < 0.05, ** *p* < 0.01, *** *p* < 0.001 and **** *p* < 0.0001.

## Results

Two clones of iPSCs were generated from dermal fibroblasts derived from a KAT6A syndrome patient who carries a *de novo* heterozygous frameshift variant (c.4025delA) in *KAT6A*, identified by exome sequencing. This variant, previously reported as part of a KAT6A syndrome cohort 14, is located in exon 17, resulting in a frameshift leading to 10 missense mutations before a premature stop codon (p.K1342Rfs*11) (Fig. S1A). Control iPSCs were generated from dermal fibroblast cells derived from the patient's healthy mother (Fig. S1A-G) as well as from a non-related healthy individual (iPSCs are characterized in Fig. S1A-G and Nayak *et al.*, 2022 31). To study the functional electrophysiological characteristics and how this pathogenic *KAT6A* variant may affect gene expression and neuron morphology, we differentiated the iPSCs into NPCs and further differentiated them into cortical neurons.

### Immature *KAT6A*-mutant cortical neurons display decreased sodium and potassium currents, increased synaptic activity and hyperexcitability

Whole-cell patch-clamp recordings were performed 4-5 weeks after the initiation of differentiation (first time point- 1^st^ tp, days 28-35) in 52 *KAT6A*-mutant cortical neurons derived from two independent iPSC clones. Recordings were also obtained from 55 control neurons derived from two healthy control lines, differentiated for the same duration.

In voltage clamp mode, spontaneous excitatory postsynaptic currents (sEPSCs) were recorded while clamping the membrane potential at -60 mV (Fig. 1A-D). *KAT6A*-mutant cortical neurons exhibited a 16-fold increased frequency of sEPSCs compared with control neurons (0.16 ± 0.06 Hz vs. 0.01 ± 0.002 Hz, respectively, *p* = 0.007, effect size = 0.502, CI = [0.36, 0.76]) (Fig. 1A-C). Additionally, a significant increase in the mean amplitude of sEPSCs was observed in the *KAT6A*-mutant neurons compared with controls (6.8 ± 0.9 pA vs. 4.0 ± 0.8 pA, respectively, *p* = 0.03, effect size = 0.45, CI = [0.045, 0.910]) (Fig. 1D). We next assessed voltage-gated sodium and potassium currents under a voltage clamp configuration (Fig. 1E-I). *KAT6A*-mutant neurons displayed significantly reduced sodium current density relative to control neurons (*p* = 9.9 x 10^-5^) (Fig. 1G). Furthermore, both slow and fast potassium current densities were significantly lower in *KAT6A*-mutant neurons compared with controls (*p* = 0.006 and *p* = 0.002, respectively) (Fig. 1H, I). *KAT6A*-mutant neurons showed significantly larger capacitance with a mean of 29.30 ± 7.10 pF compared with control neurons displaying a mean capacitance of 27.76 ± 9.60 pF (*p* = 0.049, effect size = 0.18**,** CI = [0.04, 0.36]) (Fig 1J). The membrane input conductance is shaped by ion channels, particularly inward-rectifying potassium channels and other channels active at the resting potential 37. Measurements of the input conductance showed 0.22 ± 0.04 nS for *KAT6A*-mutant neurons and 0.30 ± 0.10 nS for control neurons (*p* = 0.08, effect size = 0.26, CI = [-0.02, 0.73]) (Fig. 1K). Moreover, there were no significant differences in resting membrane potential between the two experimental groups (-44.96 ± 8.18 mV for control and -43.66 ± 10.79 mV for the patient, *p* = 0.61, effect size = 0.14, CI = [-0.26 to 0.53]) (Fig. 1L). To evaluate intrinsic excitability, we recorded evoked action potentials in current clamp mode. *KAT6A*-mutant neurons fired a significantly greater number of action potentials than control neurons in response to depolarizing current injections (52 ± 4 vs. 36 ± 3, respectively, *p* = 0.004, effect size = 0.71, CI = [0.33, 1.11]) (Fig. 1M-O). Lastly, spontaneous neuronal activity was recorded (see Experimental section) in a current clamp mode. *KAT6A*-mutant neurons displayed a marked increase in spontaneous firing relative to control neurons (12.4 ± 2.5 vs. 2.6 ± 1.2, respectively, *p* = 4.99 x 10^-4^, effect size = 0.73, CI = [0.32, 1.17]) (Fig. 1P-R).

Concurrently, calcium imaging performed at the 1^st^ tp demonstrated significant alterations in spontaneous calcium activity in *KAT6A*-mutant cortical neurons compared with controls (Fig. 2). *KAT6A*-mutant neurons exhibited a significantly higher calcium-event frequency (0.007 ± 0.009 Hz vs. 0.001 ± 0.003 Hz, *p* = 0.003, effect size = 0.75, CI = [0.24, 1.25]), indicating that more frequent spontaneous calcium events occurred (Fig. 2A). In addition, the calcium-event amplitude was significantly increased in *KAT6A*-mutant neurons (111.29 ± 20.77 vs. 97.53 ± 14.82, *p* = 0.004, effect size = 0.72, CI = [0.21, 1.22]) (Fig. 2B). Likewise, the kinetics of calcium events were also altered. *KAT6A*-mutant neurons displayed a significantly shorter rise time (0.30 ± 0.15 s vs. 0.39 ± 0.09 s, *p* = 0.004, effect size = -0.68, CI = [-1.19, -0.18]) (Fig. 2C) along with a significantly steeper rise slope (0.21 ± 0.03 ΔF/F₀/s vs. 0.19 ± 0.02 ΔF/F₀/s, *p* = 0.014, effect size = 0.61, CI = [0.11, 1.11]) (Fig. 2D). These suggest a more rapid calcium influx during spontaneous events. Additionally, *KAT6A*-mutant neurons exhibited a greater total number of calcium transients than control neurons (589 ± 209 vs. 500 ± 126, *p* = 0.04, effect size = 0.47, CI = [-0.02, 0.96]) (Fig. 2E). In contrast, calcium-transient width was comparable between the two groups (0.61 ± 0.052 s vs. 0.61 ± 0.06 s, *p* = 0.54, effect size = 0.16, CI = [-0.33, 0.65]), and no significant difference was observed in the percentage of active neurons (66.77 ± 5.30 % vs. 65.08 ± 5.96 %, *p* = 0.28, effect size = 0.30, CI = [-0.19, 0.79]) (Fig. 2F, G).

### *KAT6A*-mutant cortical neurons display decreased sodium and potassium currents as well as decreased synaptic activity and hypoexcitability at a later stage of differentiation

Whole-cell patch-clamp recordings were performed 9-10 weeks after the initiation of differentiation (second time point- 2^nd^ tp, days 63-70) in 38 *KAT6A*-mutant cortical neurons, derived from two independent iPSC clones, and 38 control neurons derived from the two control lines, differentiated for the same duration. sEPSC recordings were performed as mentioned above. We observed a 9-fold decrease in the rate of sEPSCs of the *KAT6A*-mutant cortical neurons compared with the controls (0.05 ± 0.02 Hz vs. 0.45 ± 0.12 Hz, respectively, *p* = 1.13 x 10^-6^, effect size = -0.66, CI = [-0.84, -0.46]), as shown in Fig. 3A-C. In addition, a significant decrease in the mean amplitude of the sEPSCs was observed; *KAT6A*-mutant neurons displayed a 5.7-fold smaller amplitude compared with control neurons i.e., 5.00 ± 1.10 pA vs. 28.40 ± 9.80 pA, respectively (*p* = 6.84 x 10^-7^, effect size = -0.68, CI = [-0.85, -0.48]) (Fig. 3D). We next recorded the sodium and potassium currents in a voltage clamp mode (Fig.3 E, F). We observed a significantly lower normalized sodium current in the *KAT6A*-mutant neurons compared with the control neurons (*p* = 1.36 x 10^-5^) (Fig. 3G). In addition, we observed smaller slow and fast potassium currents (normalized by the capacitance) in the *KAT6A*-mutant neurons compared with controls (*p* = 4.47 x 10^-4^, *p* = 0.004, respectively) (Fig. 3H, I). *KAT6A*-mutant neurons showed significantly lower capacitance with a mean of 27.87 ± 6.23 pF compared with control neurons displaying a mean of 33.34 ± 7.51 pF (*p* = 0.002, effect size = 0.78, CI = [-1.26, -0.31]) (Fig. 3J).

Measurements of the input conductance at this time point revealed a significantly higher input conductance for the *KAT6A*-mutant neurons compared with control neurons (0.060 ± 0.010 nS vs. 0.027 ± 0.010 nS, respectively, *p* = 0.035, effect size = 0.62, CI = [0.160, 1.095]) (Fig. 3K). However, the resting membrane potential was similar in both groups (-45.66 ± 6.35 mV for control and -47.76 ± 7.57 mV for *KAT6A*-mutant neurons, *p* = 0.22, effect size = -0.30, CI = [-0.78, 0.19]) (Fig. 3L).

To evaluate the intrinsic excitability, we recorded evoked action potentials in current clamp mode. *KAT6A*-mutant neurons fired a 3.4-fold lower number of action potentials than control neurons in response to depolarizing current injections (24 ± 3 vs. 81 ± 10, respectively, *p* = 8.03 x 10^-7^, effect size = -1.28, CI = [-1.79, -0.92]) (Fig. 3M-O). Lastly, spontaneous neuronal activity was recorded again. *KAT6A*-mutant neurons demonstrated a 13.9-fold decrease in spontaneous firing relative to control neurons (0.46 ± 0.20 vs. 6.38 ± 2.12, respectively, *p* = 0.02, effect size = -0.64, CI = [-0.95, -0.37]) (Fig. 3P-R).

In contrast to the 1^st^ tp, calcium imaging performed at the 2^nd^ tp demonstrated reduced spontaneous calcium activity in *KAT6A*-mutant cortical neurons compared with control neurons (Fig. 4). *KAT6A*-mutant neurons exhibited a significantly lower calcium-event frequency compared with control neurons (0.0035 ± 0.0025 Hz vs. 0.0058 ± 0.0019 Hz, *p* < 0.0001, effect size = -1.03, CI = [-1.50, -0.54]) (Fig. 4A). Moreover, the calcium-event amplitude was significantly reduced in *KAT6A*-mutant neurons (137.00 ± 58.01 vs. 176.02 ± 62.40, *p* = 0.015, effect size = -0.64, CI = [-1.1, -0.17]) (Fig. 4B). The total number of spontaneous calcium transients was also markedly decreased in mutant neurons (288 ± 190 vs. 466 ± 150, *p* < 0.0001, effect size = -0.61, CI = [-1.07, -0.14]) (Fig. 4C). Furthermore, calcium-transient width was significantly shorter in mutant neurons (0.66 ± 0.10 s vs. 0.73 ± 0.10 s, *p* = 0.016, effect size = -0.61, CI = [-1.07, -0.15]) (Fig. 4D), indicating briefer calcium events. In contrast, no significant differences were observed in calcium-transient rise time (0.51 ± 0.12 s vs. 0.56 ± 0.19 s, *p* = 0.27, effect size = -0.28, CI = [-0.74, 0.17]) (Fig. 4E), rise slope (0.24 ± 0.06 ΔF/F₀/s vs. 0.25 ± 0.07 ΔF/F₀/s, *p* = 0.47, effect size = -0.16, CI = [-0.62, 0.28]) (Fig. 4F), or the percentage of active neurons (66.66 ± 4.81% vs. 63.91 ± 7.04%, *p* = 0.078, effect size = 0.46, CI = [-0.002, 0.91]) (Fig. 4G).

### NPCs and cortical neurons differentiated from *KAT6A*-mutant iPSCs exhibit altered transcriptome

To identify the mechanisms underlying the altered electrophysiological characteristics of *KAT6A*-mutant cortical neurons, we undertook RNA sequencing using RNA isolated from NPCs, neurons at the 1^st^ tp of differentiation as well as neurons at the 2^nd^ tp of differentiation. Differential gene expression analysis at each time point quantified the gene expression (protein-coding genes and non-coding RNAs) fold change between *KAT6A*-mutant clones and the two control cell lines. We identified a total of 387 and 182 genes that were down- and up-regulated, respectively, in *KAT6A*-mutant NPC cells, compared with control cells (log2 fold change > ±0.5, FDR < 0.05) (Fig. 5A); 208 and 72 protein-coding genes were down- and upregulated, respectively (Fig. 5B). At the 1^st^ tp of cortical neuron differentiation, we identified 1,164 genes that were down-regulated and 984 genes that were up-regulated (Fig. 5A); 658 and 434 protein-coding genes were down- and upregulated, respectively (Fig. 5B). At the 2^nd^ tp of cortical neuron differentiation, we identified 515 genes that were down-regulated and 315 genes that were up-regulated (Fig. 5A); 293 and 159 protein-coding genes were down- and upregulated, respectively (Fig. 5B). Across all three time points of the differentiation, there were 114 common downregulated genes and 31 common upregulated genes (Fig. 5A) (of which, 61 and 13 were protein-coding genes, respectively (Fig. 5B).

Enriched KEGG pathways (FDR < 0.05) that were common to all three stages of neuron development were focal adhesion, proteoglycans in cancer, ECM-receptor interaction as well as PI3K-Akt signaling pathway (Fig. 5C, Supplementary Tables S1-3). These pathways were among the top enriched dysregulated KEGG pathways, while most of the dysregulated genes in these pathways were downregulated. We identified additional enriched signaling pathways, such as the Hippo signaling pathway, which is a common enriched pathway to both NPCs and young neurons (1^st^ tp), as well as the Wnt signaling pathway, which is a common enriched pathway to both stages of neuronal differentiation (Fig. 5C, Supplementary Tables S1-3). KAT6A was previously shown to regulate several signaling pathways including the PI3K-Akt 38, Hippo 39,40 and Wnt signaling pathway 25 via modulation of the expression of several TFs and transcriptional coregulators.

Biological process (GO:BP) enrichment analysis revealed many enriched GO terms related to tissue development and morphogenesis in all 3 stages of differentiation (Fig. 5D, Supplementary Tables S1-3). Among them were GO terms related to organ development such as neuron, bone, muscle, and heart, to name a few. Other GO terms were related to the regulation of cell motility and migration, cell adhesion, as well as regulation of signaling (Fig. 5D, Supplementary Tables S1-3). The full list of dysregulated KEGG pathways and enriched GO terms are depicted in Supplementary Tables S1-3.

Since KAT6A syndrome was previously correlated with autistic features 17,18, we compared the list of dysregulated genes in *KAT6A*-mutant cells from our RNA-seq results with the Simons Foundation Autism Research Initiative (SFARI) database. We found that many of the dysregulated genes in our RNA-seq were associated with ASD (Supplementary Tables S1-4); 27/280 (9.6%), 79/1092 (7.2%) and 31/452 (6.8%) of the dysregulated protein-coding genes in NPCs, 1^st^ tp, and 2^nd^ tp neurons, respectively, were associated with ASD. Among these genes were several TF genes, including *PAX6*, *FOXP2* and *TBX1*.

### Cortical neurons differentiated from *KAT6A*-mutant iPSCs exhibit upregulation of neural development and neuronal-related genes in the early differentiation stage

An analysis of GO-term enrichment for downregulated and upregulated genes separately revealed that the GO-BP “Nervous system development” (GO:0007399) was significantly enriched with upregulated genes in neurons at the 1^st^ tp of differentiation (78 genes, FDR = 0.038) (Fig. 6A, Supplementary Table S2). This list of genes includes genes encoding the TFs PAX6, ASCL1, MYT1L, FOXP2, IRX1, IRX2 as well as SOX1, which are key TFs in brain development 41-45. Thirteen of these 78 upregulated genes were also upregulated in neurons at the 2^nd^ tp of differentiation, including the TF genes *PAX6*, *ASCL1*, *SOX1* and *IRX1* (Supplementary Table S3).

GO:0008066, Glutamate receptor activity and GO:0004351, Glutamate decarboxylase activity, were among the GO-MF (molecular function) that were enriched with upregulated genes in neurons at the 1^st^ tp of differentiation (Supplementary Table S2). Young cortical neurons (4-5 weeks) harboring the pathogenic *KAT6A* variant exhibited upregulation of six glutamate receptor genes (*GRM3*, *GRM6* (Glutamate Metabotropic Receptor 3,6), *GRIA1*, *GRIA4* (Glutamate Ionotropic Receptor AMPA Type Subunit 1,4), *GRIN1* (Glutamate Ionotropic Receptor NMDA Type Subunit 1), and *GRIK1* (Glutamate Ionotropic Receptor Kainate Type Subunit 1)) (Fig. 6B). GABAergic neuron-related genes were also upregulated (Fig. 6B): *GAD1* and *GAD2* (which encode Glutamate Decarboxylase 1 and 2) that catalyze the production of GABA (Gamma-Aminobutyric Acid) from glutamate. *SLC6A1*, which encodes GAT-1, a GABA reuptake transporter, was upregulated. GAT-1 removes GABA from the synaptic cleft, thereby restoring it to presynaptic terminals. The GABA-B receptor *GABBR1* was upregulated as well.

Although many neuronal-related genes were upregulated in *KAT6A*-mutant cortical neurons, RNA-seq results revealed that the *SCN8A* (Sodium Voltage-Gated Channel Alpha Subunit 8/ Nav1.6), *KCNJ2* (Potassium Inwardly Rectifying Channel Subfamily J Member 2) and *CACNA1G* (Calcium Voltage-Gated Channel Subunit Alpha1 G (Cav3.1)) genes, which play a crucial role in neuronal excitability 46-48, were significantly downregulated in *KAT6A*-mutant neurons in both the 1^st^ and the 2^nd^ tp of neuron differentiation (Fig. 6C).

### Cortical neurons differentiated from *KAT6A*-mutant iPSCs display a reduced percentage of GABA-positive neurons in the early differentiation stage

We determined the percentage of GABA-positive and VGLUT1 (Vesicular glutamate transporter 1)-positive neurons in our cultures (Fig 7A-C). At the 1^st^ tp, *KAT6A*-mutant neurons exhibited a markedly lower percentage of GABA-positive neurons compared with controls (8.51 ± 0.68% vs. 18.49 ± 2.48%, *p* = 0.0046, effect size = 2.12, CI = [-15.92, -4.06]) (Fig. 7B). In contrast, the percentage of VGLUT1-positive neurons was similar between these groups (69.51 ± 1.89% vs. 67.28 ± 3.10%, *p* = 0.55, effect size = 0.27, CI = [-5.66, 10.12]) (Fig. 7C). Consistent with the selective reduction in the GABA-positive population, *KAT6A*-mutant neurons displayed a significantly higher (excitatory/inhibitory) E/I ratio compared with controls (9.00 ± 0.76 vs. 4.41 ± 0.95, *p* = 0.0017, effect size = 1.51, CI = [2.00, 7.17]) (Fig. 7D).

At the 2^nd^ tp, the percentage of GABA-positive neurons was similar between *KAT6A*-mutant and control cultures (15.55 ± 1.78% vs. 14.72 ± 0.77%, *p* = 0.67, effect size = 0.14, CI = [-3.20, 4.87]) (Fig. 7B). No significant differences were detected in the percentage of VGLUT1-positive neurons as well (70.96 ± 1.59% vs. 67.75 ± 3.01%, *p* = 0.36, effect size = 0.36, CI = [-3.93, 10.36]) (Fig. 7C), neither in the E/I ratio (5.67 ± 0.72 vs. 4.75 ± 0.30, *p* = 0.25, effect size = 0.40, CI = [-0.70, 2.55]) (Fig. 7D). Comparison across differentiation time points revealed a significant increase in the proportion of GABA-positive neurons in *KAT6A*-mutant cultures, from 8.51 ± 0.68% at the 1^st^ tp to 15.55 ± 1.78% at the 2^nd^ tp (*p* = 0.0015, effect size = 1.28, CI = [3.07, 11.02]) (Fig. 7B). In contrast, the proportion of GABA-positive neurons did not significantly change over time in control cultures (18.49 ± 2.48% vs. 14.72 ± 0.77%, *p* = 0.18, effect size = -0.76, CI = [-9.73, 2.17]).

The proportion of VGLUT1-positive neurons remained constant over time in both control cultures (67.28 ± 3.10% vs. 67.75 ± 3.01%, *p* = 0.91, effect size = 0.04, CI = [-8.64, 9.57]) and *KAT6A*-mutant cultures (69.51 ± 1.89% vs. 70.96 ± 1.59%, *p* = 0.56, effect size = 0.20, CI = [-3.60, 6.50]) (Fig. 7C).

Consistent with the increase in the GABA-positive population, the E/I ratio in *KAT6A*-mutant cultures significantly decreased from 9.00 ± 0.76 at the 1^st^ tp to 5.67 ± 0.72 at the 2^nd^ tp (*p* = 0.0034, effect size = -1.10, CI = [-5.46, -1.19]) (Fig. 7D).

The E/I ratio remained unchanged in control cultures (4.41 ± 0.95 vs. 4.75 ± 0.30, *p* = 0.74, effect size = 0.17, CI = [-1.95, 2.62]). Thus, *KAT6A*-mutant cultures displayed an early reduction in the GABA-positive neuronal population accompanied by a pronounced shift towards a higher E/I ratio, whereas these differences were no longer evident at the later differentiation stage.

### Young cortical neurons differentiated from *KAT6A*-mutant iPSCs exhibit increased neurite outgrowth, arbor complexity and soma size

To demonstrate cortical neuron differentiation, neurons at the 1^st^ tp were immunostained with the neuronal biomarker microtubule-associated protein 2 (MAP2) and CTIP2, a deep-layer cortical neuron TF marker (Fig. 8A). *KAT6A*-mutant neurons displayed a similar percentage of CTIP2-positive neurons out of MAP2-positive neurons to control neurons (Fig. 8B). To evaluate morphological differences between *KAT6A*-mutant and control cortical neurons, we quantified neurite outgrowth and arbor complexity by tracing MAP2-stained dendritic arbors (Fig. 8C-K and Supplementary Table S6) 28. MAP2 immunostaining serves as a specific biomarker for neurons, highlighting their overall neuronal presence and dendritic branching. Morphometric quantification demonstrated a marked neurite outgrowth and hyper-arborization phenotype in *KAT6A*-mutant neurons compared with control neurons. *KAT6A*-mutant neurons demonstrated a four-fold increase in total neurite length (497 ± 40 µm vs. 119 ± 11 µm, *p* = 3.25 x 10^-17^, effect size = 0.85, CI = [-459, -296]) (Fig. 8D), and a higher maximum radial extent, which indicates the Euclidean distance (straight-line distance) from the center of the cell soma to the tip of the furthest dendritic termination (54.16 ± 2.85 µm vs. 28.32 ± 1.89 µm, *p* = 3.2 x 10^-13^, effect size = 0.71, CI = [-32.56, -19.11]) (Fig. 8E). Furthermore, *KAT6A*-mutant neurons demonstrated a nearly five-fold increase in the number of branch points, which indicates higher dendritic branching (64.48 ± 5.27 vs. 11.96 ± 1.25, *p* = 6.57 x 10^-19^, effect size = 0.90, CI = [-63.19, -41.85]) (Fig. 8F), alongside increased terminal-to-primary ratio, which estimates how many terminal tips are produced per primary neurite, demonstrating higher-order arbor complexity (6.53 ± 0.37 vs. 2.82 ± 0.15, *p* = 7.52 x 10^-18^, effect size = 0.89, CI = [-4.50, -2.92] (Fig. 8G). Consistently, integrated Sholl complexity was markedly elevated for the *KAT6A*-mutant neurons (336.58 ± 24.75 intersections x μm vs. 93.48 ± 8.45 intersections x µm, *p* = 7.85 x 10^-18^, effect size = 0.88, CI = [-294.6, -191.6]) (Fig. 8H). Notably, the average branch length was similar between *KAT6A*-mutant and control neurons (7.11 ± 0.06 µm vs. 7.08 ± 0.13 µm, *p* = 0.83, effect size = 0.02, CI = [-0.32, 0.25]) (Fig. 8I). However, *KAT6A*-mutant neurites exhibited structural thickening, as evidenced by a significant increase in the mean neurite width (2.60 ± 0.05 µm vs. 1.82 ± 0.06 µm, *p* = 7.67 x 10^-23^, effect size = 1.06, CI = [-0.92, -0.63]) (Fig. 8J). Further morphological evaluation revealed increased cellular growth, evidenced by a significant enlargement of the soma area (22.67 ± 0.81 µm^2^ vs. 16.61 ± 0.68 µm^2^, *p* = 2.2 x 10^-8^, effect size = 0.54, CI = [-8.15, -3.97]) (Fig. 8K).

### *KAT6A* knock-out decreases the differentiation capacity of SH-SY5Y cells treated with retinoic acid and brain-derived neurotrophic factor

The neuroblastoma SH-SY5Y cell line is widely used as an *in vitro* model for a variety of neuropsychiatric and neurodegenerative disorders 49. Researchers utilize a fast and straightforward differentiation protocol, employing RA and BDNF, to induce the differentiation of SH-SY5Y cells into neuron-like cells that display extensive neurites and upregulated neuronal-related genes 50-52.

To further investigate the role of KAT6A in neuronal differentiation, we generated *KAT6A* knock-out (KO) SH-SY5Y clones using CRISPR/Cas9. The *KAT6A* KO clones were validated by RT-PCR (Fig. 9A) and Sanger sequencing (Fig. S2). Usually, Western blot analysis is shown for KO cells in order to demonstrate that there is no protein expression, however, there is no current commercially available KAT6A antibody that is truly specific. Sanger sequencing revealed that the *KAT6A* KO clones harbor DNA deletions that result in frameshift and premature stop codons in exons 2 and 4 of *KAT6A*. Most importantly, *KAT6A* KO did not affect the gene expression of its paralog, *KAT6B* (Fig. 9B).

In order to differentiate SH-SY5Y cells into neuronal-like cells, SH-SY5Y WT (Wild type) and *KAT6A* KO cells were treated with RA in reduced serum medium (1% FBS) for 5 days, followed by BDNF treatment in medium without serum for an additional 48 hours (Fig. 9C, D). WT cells treated with RA displayed decreased cell clustering along with extension of neurites, a typical neuronal phenotype 51,52. BDNF treatment enhanced this neuronal phenotype, and WT cells accordingly exhibited an extensive network of neurites (Fig. 9C, D). In contrast, the *KAT6A* KO clones displayed reduced differentiation capacity. *KAT6A* KO cells treated with RA were less dispersed than WT cells and did not form an extensive network of neurites as their WT cell counterparts did, following treatment with BDNF (Fig. 9C, D).

### *KAT6A* KO alters gene expression in undifferentiated and differentiated SH-SY5Y cells

To further evaluate the impact of *KAT6A* KO on SH-SY5Y cell differentiation capacity, we quantified neuronal markers that were previously shown to be upregulated in SH-SY5Y cells following differentiation with RA and BDNF 53,54, using RT-PCR. WT cells displayed a significant increase in the gene expression of *NTRK2*, the BDNF receptor TrkB, in differentiated cells, when compared with cells treated with DMSO as a control (Fig. 10A). The increase in the expression of *NTRK2* indicates that the cells were differentiated into neuron-like cells. *NTRK2* expression increased in RA-treated *KAT6A* KO cells, compared with *KAT6A* KO cells treated with DMSO as a control, but was much lower than in WT cells (accounting for only ~45-fold increase as compared with the ~500-fold increase in WT cells; *p* = 0.006 for both *KAT6A* KO C1 and C2 clones). BDNF treatment did not affect *NTRK2* expression in WT cells, but further increased *NTRK2* expression in the *KAT6A* KO clones by ~2.6-fold (accounting for 23% of WT expression level, *p* = 0.01 for both *KAT6A* KO C1 and C2 clones). Gene expression of additional neuronal biomarkers, *PSD95*, *SYP* and *ENO2* was elevated only in WT cells but not in *KAT6A* KO cells (Fig. 10B-D).

We also determined the gene expression of *KAT6A* as well as *KAT6B* following differentiation with RA and BDNF. *KAT6A* gene expression did not change in WT cells following RA and BDNF treatment (Fig. 10E). However, *KAT6B* expression was elevated in WT cells after treatment with RA, by 1.6-fold (*p* = 0.049), whereas *KAT6B* was also upregulated in *KAT6A* KO cells, but to a lower extent (55% and 60% of expression in *KAT6A* KO cells compared with WT cells after RA treatment, respectively; *p* = 0.007, 0.005 for *KAT6A* KO C1 and C2 clones, respectively) (Fig. 10F). Using RT-PCR, the quantification of *KAT6B* expression levels in NPCs differentiated from iPSCs revealed that *KAT6B* mRNA levels were similar between *KAT6A*-mutant and control NPCs (Fig. 10G), consistent with the results obtained with undifferentiated SH-SY5Y cells (Fig. 9B). In sharp contrast, *KAT6B* gene expression in *KAT6A*-mutant young neurons was markedly upregulated compared with control neurons (Fig. 10G), being similar to the elevation of *KAT6B* following differentiation of SH-SY5Y cells (Fig. 10F).

To further decipher the impact of KAT6A deficiency on gene expression in *KAT6A*-mutant cells, we utilized RT-PCR to quantify gene expression and identify genes that are dysregulated in both cells that were differentiated from *KAT6A*-mutant iPSCs, as well as in *KAT6A* KO SH-SY5Y clones. We found that *RSRC1*, *CRABP2* and *SHOX2* were downregulated in *KAT6A*-mutant NPCs as well as in *KAT6A* KO SH-SY5Y cells (Fig. 10H-M). RSRC1, a member of the serine and arginine rich-related protein family, has a role in alternative splicing and transcription regulation 55. RNA-seq results of NPCs showed a marked downregulation of *RSRC1* expression in *KAT6A*-mutant cells compared with control cells (log_2_ fold change = -3) (Fig. 10H). *RSRC1* expression was also downregulated in *KAT6A* KO SH-SY5Y clones C1 and C2, to 66% (*p* = 2.97 x 10^-5^) and 67% (*p =* 1.29 x 10^-4^), respectively, compared with WT cells) (Fig. 10I).

The *CRABP2* gene, which was shown to be upregulated upon RA treatment, encodes the cellular retinoic acid-binding protein 2, which binds to and shuttles RA from the cytoplasm to the nucleus, where RA regulates gene expression 54,56. We found that *CRABP2* was also downregulated in *KAT6A*-mutant NPCs as well as in *KAT6A* KO SH-SY5Y cells. RNA-seq results of NPCs showed *CRABP2* downregulation in *KAT6A*-mutant cells (log_2_ fold change = -2.54) compared with control cells (Fig. 10H). *CRABP2* expression was also downregulated in *KAT6A* KO SH-SY5Y clones C1 and C2, to 66% (*p* = 0.02) and 55% (*p* = 0.01), respectively, compared with WT cells (Fig. 10J). In agreement with previous reports 56, *CRABP2* gene expression was elevated following RA treatment (Fig. 10K). Remarkably, *CRABP2* expression was elevated in WT SH-SY5Y cells by 277-fold compared with control cells treated with DMSO, whereas its expression increased in RA-treated *KAT6A* KO clones, compared with *KAT6A* KO cells treated with DMSO as a control, albeit was much lower than in WT cells (accounting for only ~30% increase as compared with the increase of the WT cells; *p* = 0.0001/0.002 for *KAT6A* KO C1 and C2 clones, respectively).

Lastly, the homeobox TF SHOX2 was highly downregulated in *KAT6A*-mutant NPCs (log_2_ fold change = -5) (Fig. 10H). We also observed a 2-fold decrease in *SHOX2* expression in *KAT6A* KO SH-SY5Y clones C1 and C2, (*p* = 2.5 x 10^-5^ and *p =* 4.4 x 10^-5^, respectively, compared with WT cells) (Fig. 10L). Furthermore, when WT SH-SY5Y cells were differentiated with RA and BDNF, *SHOX2* expression was increased by 2-fold (*p* = 0.02), whereas *SHOX2* expression remained 2-fold lower in *KAT6A* KO SH-SY5Y clones C1 and C2 treated with RA and BDNF, compared with WT cells (*p* = 0.03 and 0.02, respectively) (Fig. 10M).

## Discussion

Herein, we report on the generation of iPSCs from a KAT6A syndrome patient and healthy controls, enabling us to establish and characterize the first *in vitro* model of iPSC-derived *KAT6A*-mutant NPCs and cortical neurons. The cerebral cortex and its cortical neurons are of paramount importance for higher-level brain functions; these include memory, thinking, learning, reasoning, perception, problem solving, emotions, awareness and consciousness 57-59. Furthermore, motor cortex neurons initiate voluntary motor functions. Since both categories of activity have been reported to be impaired in KAT6A syndrome patients 18, we chose to differentiate the above NPCs into cortical neurons. Using a whole-cortical neuron cell patch-clamp, electrophysiological recordings revealed a hyperexcitability pattern in young *KAT6A*-mutant neurons differentiated for 4-5 weeks. Furthermore, *KAT6A*-mutant neurons displayed a reduced percentage of GABA-positive cells at this time point, leading to an increased E/I ratio. In contrast, cortical neurons differentiated for 9-10 weeks exhibited hypoexcitability when compared with cortical neurons from healthy individuals. The calcium-imaging findings are consistent with the electrophysiological recordings obtained at both developmental stages. Specifically, the increased frequency, larger amplitude, and faster kinetics of spontaneous calcium events at the 1^st^ tp indicate enhanced spontaneous activity in *KAT6A*-mutant cortical neurons. These results accord with the patch-clamp recordings, which demonstrated increased spontaneous firing, elevated spontaneous synaptic activity, and enhanced intrinsic excitability. Finally, the identical proportion of active neurons suggests that the mutation alters the functional properties of existing active neurons, rather than shifting the total fraction of neurons contributing to network activity. Remarkably, early maturation, hyperexcitability, and a reduced percentage of GABA-positive neurons are phenotypes that were previously observed in young (differentiated for 3-5 weeks) cortical neurons derived from iPSCs harboring pathogenic variants in genes underlying neurodevelopmental disorders with a high prevalence of ASD, including *GRIN2B*, *SHANK3*, and *UBTF* 29. Likewise, a similar phenotype was also observed in additional cases of neurons differentiated from iPSCs obtained from patients with ASD or with ID and epilepsy syndromes 28,30,60.

Our *KAT6A*-mutant cortical neurons were less excitable than healthy control neurons at a later stage of the differentiation, resembling neurons derived from an ASD patient carrying an *IQSEC2* pathogenic variant 30. Unexpectedly however, although our *KAT6A*-mutant cortical neurons exhibited early hyperexcitability which was later followed by hypoexcitability, we observed substantially decreased sodium and potassium currents in both early and late stages of neuronal differentiation. This is in sharp contrast to the increased sodium currents in young cortical neurons differentiated from iPSCs derived from patients harboring pathogenic variants in ASD related genes 29,30.

To gain mechanistic insight into the molecular basis of this early excitability yet decreased sodium and potassium currents, we undertook RNA-seq analysis. This revealed that the sodium voltage-gated channel gene *SCN8A* was downregulated in *KAT6A*-mutant cortical neurons, possibly accounting for the decreased sodium currents. *SCN8A* encodes a member of the sodium channel α subunit gene family that is abundantly expressed in neurons of the central nervous system (CNS) and plays a critical role in regulating neuronal excitability 61. LoF pathogenic variants in *SCN8A* leading to a decrease in sodium current and neuronal firing are associated with ASD, ID and motor impairment 46,61-63.

Potassium ion channels are also crucial for cellular excitability. Remarkably, *KCNJ2*, which encodes the potassium inwardly rectifying channel Kir2.1, was markedly downregulated in differentiated *KAT6A*-mutant cortical neurons. It was previously shown that overexpression of *KCNJ2* in various neuron types leads to decreased neuronal excitability 64-67. Kir2.1 channels play a key role in maintaining a stable resting membrane potential (RMP), thereby regulating neuronal firing. As *KCNJ2* was markedly downregulated in our *KAT6A*-mutant cortical neurons, the low levels of Kir2.1 channels necessarily result in a diminished inward potassium current and possibly a less negative RMP, rendering neurons more prone to depolarization and firing action potentials. Intriguingly, our findings revealed that the RMP remained unchanged in *KAT6A*-mutant neurons, indicating that an alternative mechanism must account for this increased cellular excitability. Furthermore, although not statistically significant, neurons derived from the KAT6A syndrome patient exhibited reduced input conductance compared with control neurons at the 1^st^ tp, which can reflect altered channel activity. While a decrease in input conductance often coincides with shifts in RMP due to altered leak currents, our findings demonstrate a stable RMP alongside decreased conductance at the 1^st^ tp. This suggests that Kir2.1 downregulation may trigger compensatory adjustments in other background leak channels, thereby maintaining electrochemical balance, while altering overall membrane excitability. Interestingly, this pattern was reversed at the 2^nd^ tp, where patient-derived neurons displayed higher input conductance relative to control neurons. This transition aligns precisely with the phenotype we observed, i.e. hyperexcitability and decreased potassium current in the early *KAT6A*-mutant cortical neurons followed by hypoexcitability in the 2^nd^ tp. Interestingly, previous studies have shown that increased excitability can contribute to various neurological conditions, including epilepsy, ASD, ataxia, and ID 68,69. Hyperexcitability was also shown to elicit excitotoxicity that may lead to neuronal damage or cell death and is associated with neurodegenerative diseases and ASD 70-72.

Of special note is our finding via MAP2 immunostaining, that *KAT6A*-mutant cortical neurons exhibit early maturation, represented by increased spatial coverage, dendritic arbor complexity, neurite width and soma size, compared with their healthy counterparts. These remarkable morphometric features are likely a result of differential gene expression, as previously observed in iPSC-derived neurons from ASD patients 28. This further supports the electrophysiological findings showing that the *KAT6A*-mutant neurons mature faster than control neurons 73. The major morphometric alterations detected in our precociously differentiated *KAT6A*-mutant neurons may have inflicted a highly distinct electrophysiological activity than normal neurons. In this respect, dendritic abnormalities are constantly found in ASD and neurodevelopmental disorders associated with ID 28,74-77, including neurodevelopmental disorders associated with pathogenic variants in *CREBBP* and *EP300*, encoding the homologous CBP and p300 lysine-acetyltransferases 78. Neurons differentiated from iPSCs derived from Rubinstein-Taybi Syndrome (RSTS) patients, carrying pathogenic variants in *CREBBP*- and *EP300*, exhibited altered morphology of young neurons (differentiated for 6 weeks), and hypoexcitability after 10 weeks of differentiation 78, similar to our results with *KAT6A*-mutant neurons. Dendritic anomalies reported for ID disorders mostly included decreased dendrite length/branching or decreased spine density 75,76. RSTS neurons displayed a higher branch number, however their mean branch length was significantly decreased 78.

Interestingly, similarly to our findings, neural differentiation of iPSCs derived from cardiofaciocutaneous (CFC) syndrome patients showed early maturation of cortical glutamatergic neurons 79. CFC patients commonly display developmental delay, hypotonia and motor delay, epilepsy, ID, increased risk for ASD, seizures, and a higher incidence of structural brain abnormalities 79,80. CFC iPSCs carried a gain-of-function pathogenic variant in *BRAF* (B-Raf Proto-Oncogene, Serine/Threonine Kinase), which we found to be upregulated in our *KAT6A*-mutant young neurons (Supplementary Table S2). BRAF plays a role in regulating the MAP kinase/ERK signaling pathway, which affects cell division and differentiation 81. Ras/MAPK pathway signaling is a major regulator of neurodevelopment. Its dysregulation by pathogenic variants in genes that encode protein components of the pathway leads to various neurological disorders collectively called RASopathies 80,81. A previous study suggested that NPCs carrying a pathogenic variant in *BRAF* were leaving the mitotic progenitor state earlier than controls 79. Furthermore, neurons differentiated from the CFC NPCs displayed a higher number of neurites, which were also longer.

Evidence that identifies the neural progenitor cell stage as the critical developmental period affecting the accelerated differentiation of neurons comes from recent studies 28,82. Ciceri *et. al.,* revealed that the epigenetic profile of NPCs serves as a rate-limiting factor that determines the pace of neuronal maturation by delaying the expression of maturation genes 82. This repression is gradually alleviated as differentiation progressively proceeds, allowing for the timely expression of maturation-specific genes. Alteration of this molecular program in NPCs, following the inhibition of several histone lysine methyltransferases, resulted in an accelerated maturation of cortical neurons 82. Furthermore, Schafer *et. al.,* found changes in chromatin accessibility in ASD neural stem cells, and direct differentiation of ASD iPSCs into induced cortical neurons abolished ASD-associated phenotypes in the differentiated neurons 28. Given its role as a histone acetyltransferase, KAT6A may function as a key epigenetic regulator which directly or indirectly regulates the timely expression of neuronal maturation genes.

In contrast to the early maturation of neurons differentiated from *KAT6A*-mutant iPSCs, we find that *KAT6A* KO impaired the differentiation of human SH-SY5Y cells. However, one should bear in mind that the patient-derived *KAT6A*-mutant iPSCs that we studied here are in fact heterozygous for the pathogenic variant and therefore are not completely deficient in KAT6A. While a reduction in KAT6A dosage as in heterozygotes may relieve transcriptional repression or prematurely trigger developmental gene programs to accelerate maturation, a complete KO likely causes widespread transcriptional failure that impairs differentiation and other cellular functions. Neural differentiation requires tight regulation of gene expression, therefore, when gene expression is impaired, it could lead to premature differentiation or even inhibition of differentiation, both of which impair neurodevelopment 7. Epigenetic regulators, such as histone acetyltransferases and histone deacetylases, maintain epigenetic control of lineage-specific gene transcription, and their expression is required for proper neural development 83,84. Precise regulation of H3K9 acetylation was found to be crucial for neural development 83. The authors showed that the level of H3K9 changes along with the *in vitro* neural differentiation of human ESCs. During the first 4 days of the differentiation, it decreases, and then increases during days 5-8, shifting from H3K9 acetylation at pluripotency genes' loci to H3K9 acetylation at neurodevelopmental genes' loci. In this respect, both KAT6A and KAT6B were found to acetylate histone residue H3K9 at the promoters of specific genes 4,12,19,24,85-90. The complete loss of KAT6B in mice led to a global decrease in acetylated H3K9 and resulted in impaired cortex development 85,91. *Kat6b* KO in mouse embryonic neural stem and progenitor cells attenuated self-renewal as well as neuronal differentiation and neurite outgrowth, whereas overexpression of *Kat6b* resulted in increased neuronal differentiation from neural stem cells and increased the number of neurons, demonstrating the significant role of KAT6B in neuronal development 85,87,92. These results are consistent with the increased expression of *Kat6b* during cortical neurogenesis in mice 85,91. *KAT6B* elevation following the differentiation of SH-SY5Y cells implies that KAT6B is directly involved in neuronal differentiation of SH-SY5Y cells and supports previous data suggesting that KAT6B has a role in neural development as stated above. *KAT6B* was also upregulated in our young *KAT6A*-mutant neurons, supporting our evidence of their early maturation.

Consistent with the early maturation of *KAT6A*-mutant cortical neurons, they exhibited an early upregulation of many genes related to neuronal development and differentiation, including several key TFs vital for neuronal lineage fate and differentiation, including SOX1, PAX6, FOXP2, MYT1L, ASCL1 and TBX1 93. In accord with our present findings, *Kat6a*^+/-^ mice display upregulation of neuronal development and maturation genes, including axon guidance and synapse organization-related genes, in the developing dorsal telencephalon at E12.5 26. These findings lend further support to our observations, suggesting that downregulation of KAT6A accelerates neurons' maturation. Interestingly, some of the upregulated TFs in our *KAT6A*-mutant cortical neurons, including PAX6, FOXP2, MYT1L and TBX1 are associated with ASD (Supplementary Tables S1-4). SOX1 plays a direct role in neuronal lineage commitment and differentiation and its overexpression in cultured neural progenitor cells is sufficient to induce neuronal lineage commitment 44,94. PAX6, which was among the elevated TFs in our current study, is a key TF that regulates the expression of many genes implicated in brain development, many of which are ASD-associated genes 41,95-97. PAX6 maintains neural stem cells and progenitor cells while also promoting neuronal differentiation, generation and migration of neurons 98. PAX6 interacts with FOXP2 41, which was also upregulated in our young *KAT6A*-mutant neurons. FOXP2 was found to regulate neurogenesis during embryonic cortical development and plays a major role in language development 99. Pathogenic variants in *FOXP2* or dysregulation of its gene expression are associated with ASD and language impairment 41,93,100. The combination of ASCL1, POU3F2 (BRN2) and MYT1L expression was shown to be sufficient to reprogram fibroblasts and other somatic cells to induce neuronal cells 42. Both *ASCL1* and *MYT1L* were upregulated in young *KAT6A*-mutant neuron cells. MYT1L expression is regulated by PAX6 41. Along this line, TBX1 was previously shown to play a key role in cortical neurogenesis 101. Indeed, disruption of the TBX1 gene in mice resulted in altered neuronal differentiation and polarity as well as impaired cortical lamination. Collectively, the above findings lend strong support to the molecular mechanisms underlying altered cortical differentiation and function. The model of TF-driven developmental acceleration is highly convergent with a recent study by Wang *et al.* 102, revealing that multiple ASD-associated variants rewire convergent protein interaction networks to drive neurodevelopmental pathology. Specifically, the authors demonstrated that high-confidence ASD risk proteins systematically converge on physical networks enriched within NPCs of the excitatory lineage, and that patient-derived missense *FOXP1* variants selectively disrupt FOXP1 interaction with FOXP4. This disruption causes a gain-of-function redistribution of FOXP4 to ectopic genomic sites, directly driving premature cortical neuron differentiation and altered neural activity in human brain organoids. It is highly plausible that in a similar manner to these ASD network-rewiring variants, the heterozygous loss of *KAT6A* also drives precocious maturation by altering the physical interactions and genomic targeting of central transcriptional networks. Supporting this convergent network hypothesis is our finding of early upregulation of key TFs, some of which are associated with ASD, including FOXP2. These findings suggest that epigenetic disruption of the KAT6A chromatin landscape may directly perturb the precise transcriptional balance and physical interactome of the abovementioned differentially expressed TFs, and specifically the FOXP family, during early cortical neurogenesis.

Our finding that *CRABP2*, *RSRC1* and *SHOX2* were downregulated in both *KAT6A*-mutant NPCs and *KAT6A* KO SH-SY5Y cells suggests that KAT6A regulates the expression of these key genes, either directly or indirectly. RA binds to CRABP2, following which the latter shuttles it to the nucleus, where it regulates the transcription of key developmental genes 103. In our current study, SH-SY5Y cell differentiation was induced by RA, therefore, *CRABP2* downregulation is likely to underly their impaired differentiation capacity. Importantly, RA signaling pathways were found to be involved in neuronal differentiation 103-106, hence, *CRABP2* downregulation presumably negatively affected the expression of neuronal genes in *KAT6A*-mutant NPCs.

Another interesting gene that was downregulated in *KAT6A*-mutant NPCs and *KAT6A* KO SH-SY5Y cells is *RSRC1*. In this respect, *KAT6A* KO MOLM-13 AML cells exhibited downregulation of H3K9ac on the RSRC1 gene 107. These data suggest that *RSRC1* may be a KAT6A target gene. Genetic LoF pathogenic variants in RSRC1, which plays a role in alternative splicing, were found to be associated with an autosomal recessive syndrome of ID, accompanied by aberrant behavior, hypotonia and mild facial dysmorphism 55. Using long-read RNA sequencing (Iso-Seq), Nava et al., reported dysregulated isoform usage in *KAT6A*-mutant cerebral organoids 108. Among these, seven ASD risk genes displayed a significant isoform-specific dysregulation, including *EPHB2* and *CACNA1G*, which are also known as epilepsy-related genes. The authors concluded that this isoform-specific dysregulation highlights the impact of pathogenic *KAT6A* variants on alternative splicing and neural development.

Yan and colleagues 107 found that KAT6A binds to the chromatin of the TF *SHOX2*, which is downregulated following *KAT6A* KO, and is associated with H3K9ac downregulated regions following *KAT6A* KO in human MOLM-13 AML cells. These results indicate that KAT6A directly regulates *SHOX2* gene expression in AML cells and possibly in other cell types. The *SHOX2* gene, which is located adjacent to the *RSRC1* gene on chromosome 3, is a homeobox gene that is essential for the development of multiple organs, including limb, heart, craniofacial compartments, as well as neurons and brain 109-114. SHOX2 is expressed in embryonic and mature CNS, and evidence suggests that it may be important to brain function as well. Using conditional KO animals it was recently 114 demonstrated that SHOX2 is important for neuron firing capacities, likely by affecting the expression of mRNAs of multiple ion channels, including *CACNA1G* (Cav3.1), which was consistently downregulated in our *KAT6A*-mutant cortical neurons. As described above, a recent study showed that the *CACNA1G* gene exhibited a significantly reduced usage of a specific RNA isoform in cerebral organoids differentiated from KAT6A-patients' iPSCs 108. In this respect, *CACNA1G* encodes a subunit of a T-type, low-voltage activated calcium channel, which regulates neuronal excitability. Importantly, pathogenic variants in genes encoding for Cav3 channels, including *CACNA1G*, *CACNA1H*, and *CACNA1I,* have been linked to an assortment of neurodevelopmental, neurological, and psychiatric disorders known as neuronal Cav3 channelopathies 47. Consistently, *Cacna1g* KO mice display impaired motor performance and cerebellar learning 47. Along this vein, pathogenic variants in the *CACNA1G* gene, as well as monoallelic deletions, were linked to ID disorders 47,115-117.

In summary (Fig. 11), we conclude that the pathogenic *KAT6A* variant induces premature maturation of differentiated cortical neurons. This is supported by our novel findings that, compared with control neurons, young *KAT6A*-mutant neurons express high levels of multiple key neuronal development genes, exhibit extended spatial coverage and neurite arborization, and display hyperexcitability. The premature hyperexcitability at an early stage of neuron development possibly leads to increased neuronal vulnerability and excitotoxicity. At a later differentiation stage, downregulation of key ion channel genes that leads to decreased sodium and potassium currents, result in neuron hypoexcitability, presumably accounting for various behavioral, intellectual and physical disabilities.

## Supplementary Material

Supplementary figures, table legends and Supplementary Table 5.

Supplementary table 1.

Supplementary table 2.

Supplementary table 3.

Supplementary table 4.

Supplementary table 6.

## Figures and Tables

**Figure 1:**
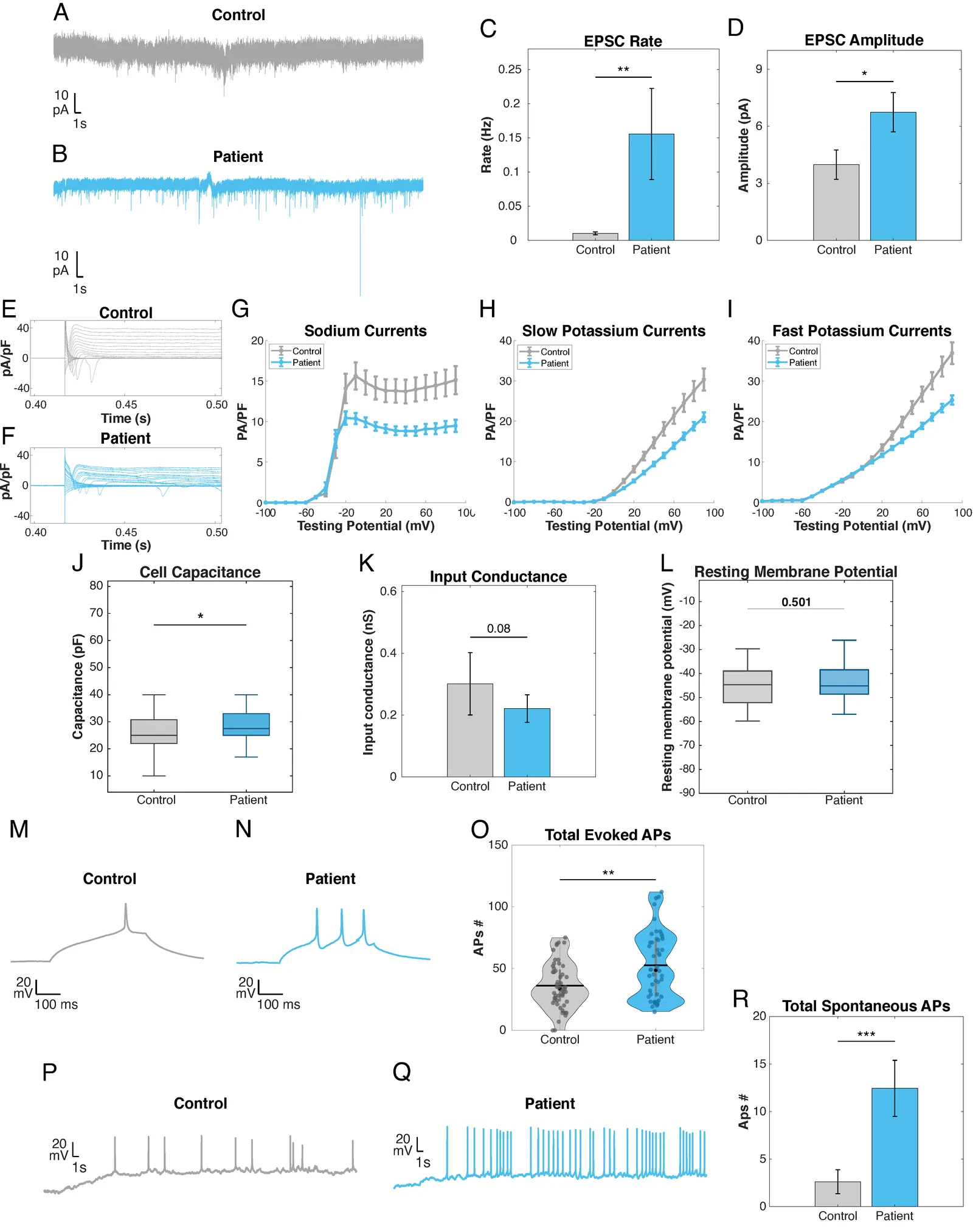
** Young (i.e., following 4-5 weeks of differentiation) *KAT6A*-mutant cortical neurons display hyperexcitability yet diminished sodium and potassium currents.** A, B) Representative traces of sEPSCs measured in control (A) and *KAT6A*-mutant (B) cortical neurons. C) The mean rate of synaptic events is higher in *KAT6A*-mutant neurons compared with control neurons (*p* = 0.007). D) The mean amplitude of sEPSCs is increased in *KAT6A*-mutant neurons (*p* = 0.03). E, F) Representative traces of sodium and potassium currents recorded in a voltage-clamp mode in control (E) and *KAT6A*-mutant (F) neurons. G) Mean sodium currents in *KAT6A*-mutant neurons are decreased compared with control neurons (*p* = 9.9 x 10^-5^). H) Mean slow potassium currents in *KAT6A*-mutant neurons are decreased compared with control neurons (*p* = 0.006). I) Mean fast potassium currents are decreased in *KAT6A*-mutant compared with control neurons (*p* = 0.002). J) Cell capacitance is increased in *KAT6A*-mutant neurons (*p* = 0.049). K) Input conductance of *KAT6A*-mutant neurons compared with control neurons (*p* = 0.08). L) No significant difference in resting membrane potential was observed between the two groups (*p* = 0.501). M, N) Representative recordings of evoked action potentials in a current-clamp mode of a control (M) and a *KAT6A*-mutant (N) neuron. O) The total number of evoked action potentials is higher in *KAT6A*-mutant neurons compared with control neurons (*p* = 0.004); (The black line represents the mean and the black dot represents the median). P, Q) Representative recordings of spontaneous action potentials in a current-clamp mode of a control (P) and a *KAT6A*-mutant (Q) neuron. R) The total number of spontaneous action potentials is higher in *KAT6A*-mutant neurons compared with control neurons (*p* = 4.99 x 10^-4^). Error bars represent the standard error; control (n = 55 neurons), KAT6A (n = 52 neurons). In this figure and the following figures, asterisks represent statistical significance as follows: *p < 0.05, ** *p* < 0.01, *** *p* < 0.001 and **** *p* < 0.0001.

**Figure 2:**
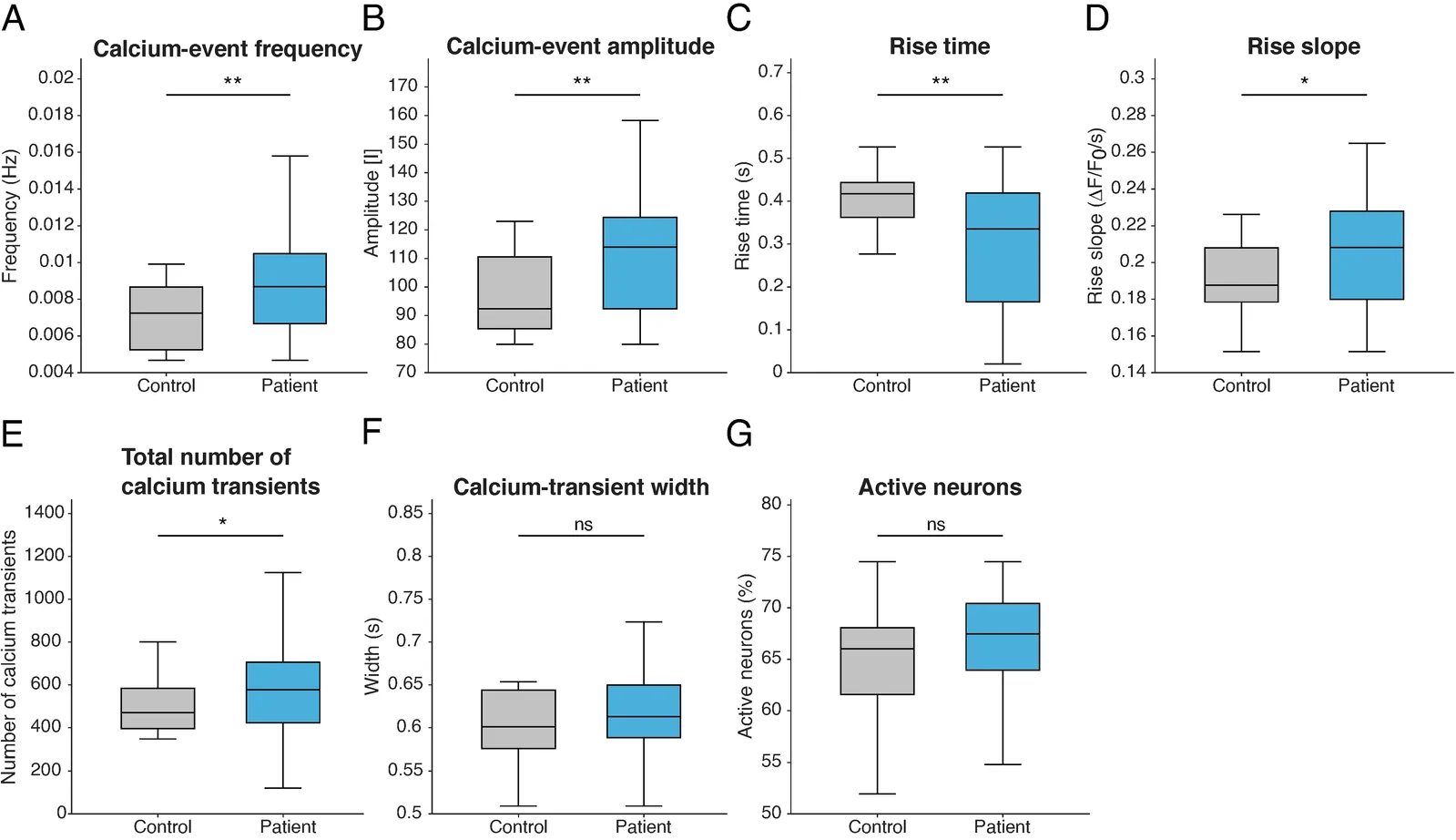
** Young (i.e., following 4-5 weeks of differentiation) *KAT6A*-mutant cortical neurons display increased spontaneous calcium activity.** Calcium-event frequency (A) and amplitude (B) are significantly higher in *KAT6A*-mutant neurons (*p* = 0.003, *p* = 0.004, respectively) Mutant neurons display a significantly shorter rise time (*p* = 0.004 (C), a steeper rise slope (*p* = 0.014) (D), and a higher total number of calcium transients (*p* = 0.041) (E). Calcium-transient width (F) and percentage of active neurons (G) are not significantly changed (*p* = 0.54, *p* = 0.28, respectively). Data are presented as mean ± SEM. ns, not significant.

**Figure 3:**
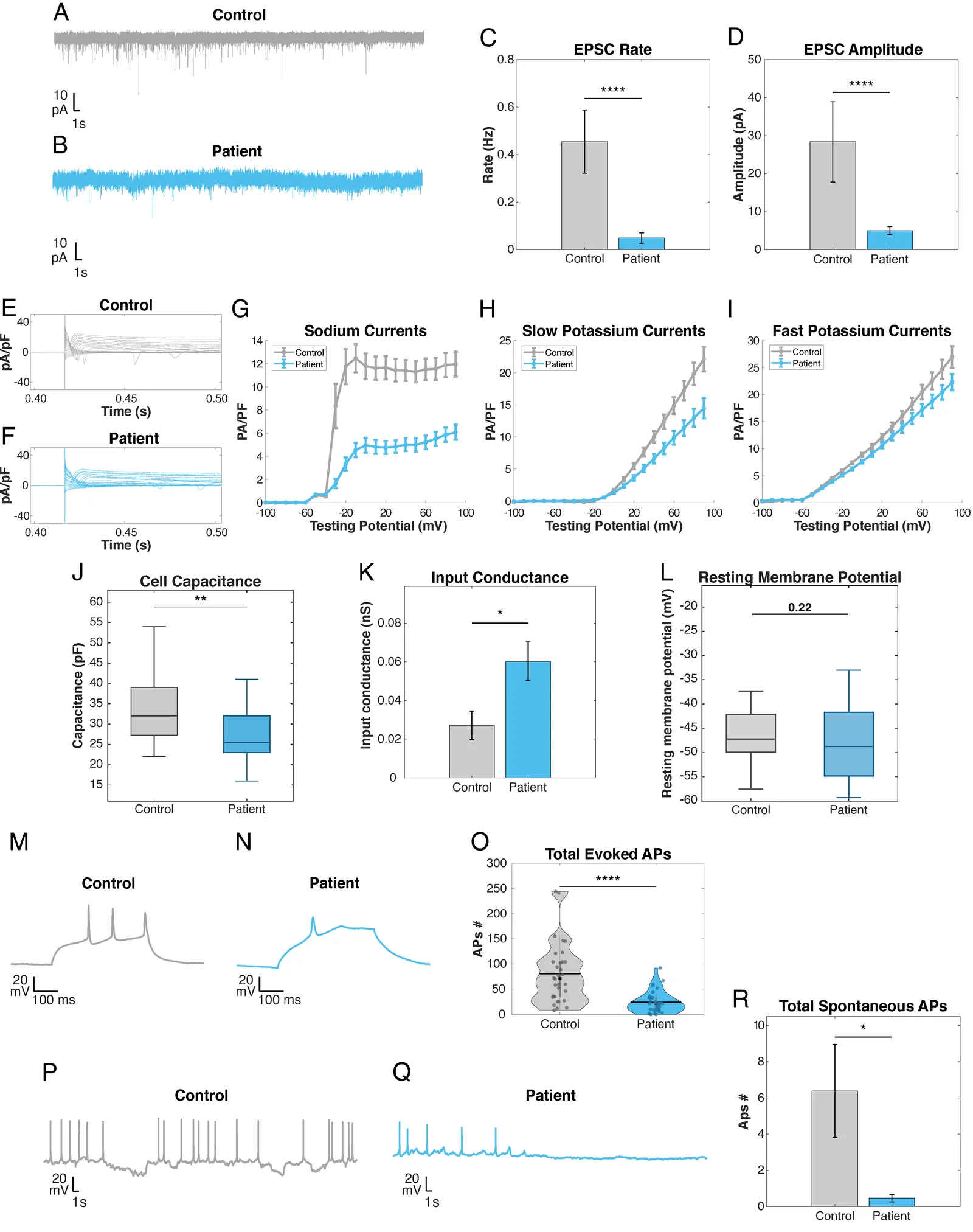
** Following 9-10 weeks of differentiation, *KAT6A*-mutant cortical neurons display hypoexcitability and exhibit reduced sodium and potassium currents.** A, B) Representative traces of sEPSCs measured in control (A) and *KAT6A*-mutant (B) cortical neurons. C) The mean rate of synaptic events is significantly decreased in *KAT6A*-mutant neurons compared with control neurons (*p* = 1.13 x 10^-6^). D) The mean amplitude of sEPSCs is significantly decreased in *KAT6A*-mutant neurons (*p* = 6.83 x 10^-7^). E, F) Representative traces of sodium and potassium currents recorded in a voltage-clamp mode in control (E) and *KAT6A*-mutant (F) neurons. G) Mean sodium currents in *KAT6A*-mutant neurons are decreased compared with control neurons (*p* = 1.36 x 10^-5^). H) The mean slow potassium currents in *KAT6A*-mutant neurons are decreased compared with control neurons (*p* = 4.47 x 10^-4^). I) The mean fast potassium currents are decreased in *KAT6A*-mutant neurons compared with control neurons (*p* = 0.004). J) Cell capacitance is decreased in *KAT6A*-mutant neurons (*p* = 0.002). K) Input conductance of *KAT6A*-mutant neurons is increased compared with control neurons (*p* = 0.035). L) Resting membrane potential did not significantly differ between the two experimental groups (*p* = 0.22). M, N) Representative recordings of evoked action potentials in a current-clamp mode of a control (M) and a *KAT6A*-mutant (N) neuron. O) The total number of evoked action potentials is significantly lower in *KAT6A*-mutant neurons compared with control neurons (*p* = 8.03 x 10^-7^); (The black line represents the mean, and the black dot represents the median). P, Q) Representative recordings of spontaneous action potentials in a current-clamp mode of a control (P) and a *KAT6A*-mutant (Q) neuron. R) The total number of spontaneous action potentials is significantly lower in *KAT6A*-mutant neurons compared with control neurons (*p* = 0.02). Error bars represent the standard error; control (n = 38 neurons), KAT6A (n = 38 neurons).

**Figure 4:**
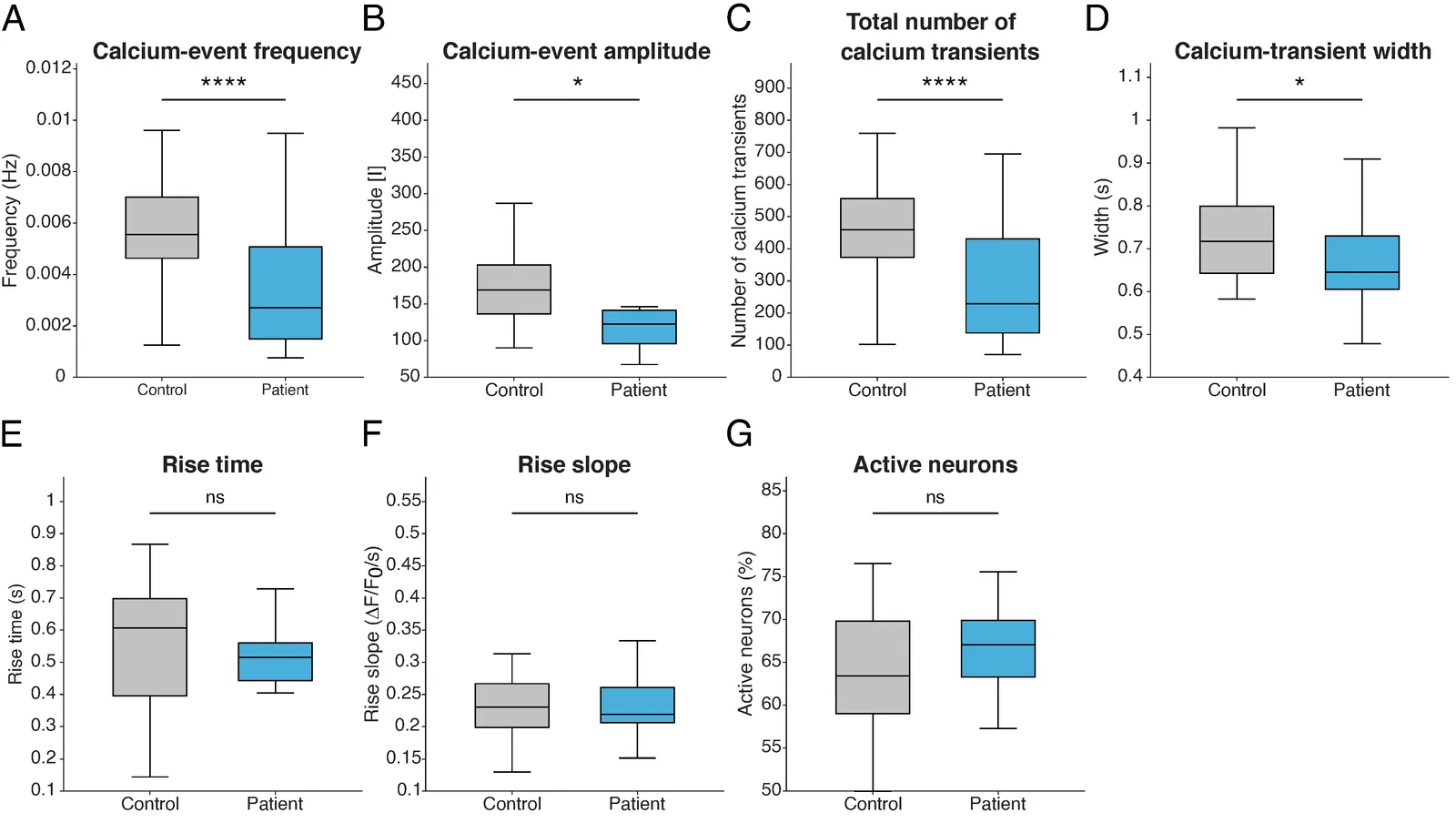
** Following 9-10 weeks of differentiation, *KAT6A*-mutant cortical neurons display decreased spontaneous calcium activity.** Calcium-event frequency (A) and amplitude (B), as well as total number of calcium transients (C), and calcium-transient width (D), are significantly decreased in *KAT6A*-mutant neurons (*p* < 0.0001, *p* = 0.015, *p* < 0.0001, *p* = 0.016, respectively). In contrast, rise time (E), rise slope (F), and the percentage of active neurons (G) are not significantly changed (*p* = 0.27, *p* = 0.47, *p* = 0.078, respectively). Data are presented as mean ± SEM. ns, not significant.

**Figure 5:**
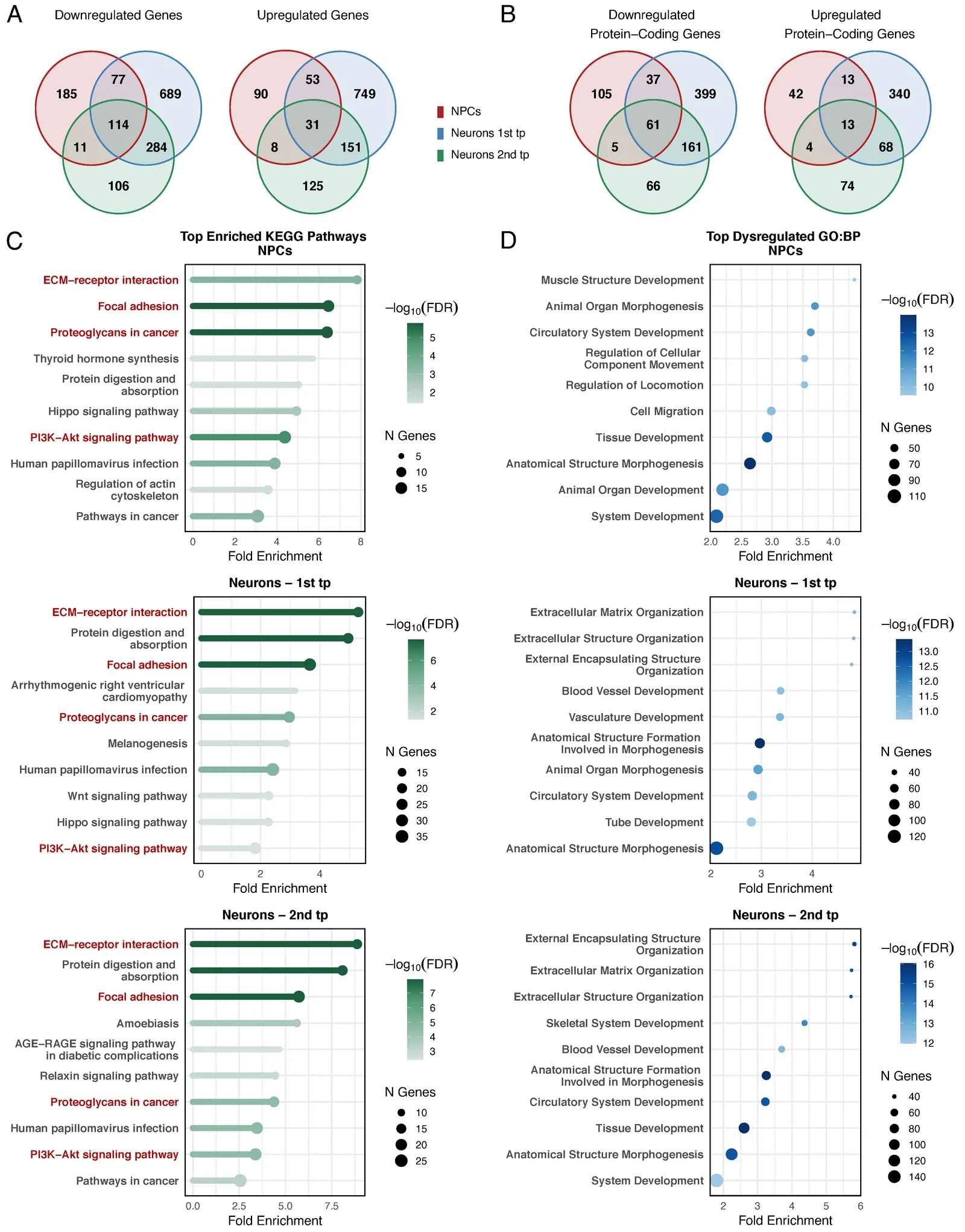
** Transcriptome alterations in NPCs and cortical neurons differentiated from iPSCs derived from a KAT6A patient.** (A, B) Gene overlap analysis. A) Venn diagrams of overlapping down-regulated and up-regulated genes among NPCs, cortical neurons at the 1^st^ tp, and the 2^nd^ tp of differentiation, respectively. B) Same as (A) for protein-coding genes only. C) Significantly enriched KEGG pathways in NPCs and cortical neurons differentiated from KAT6A patient's and controls' iPSCs. Dysregulated pathways that were common to all three stages of differentiation are marked in red. D) The top dysregulated GO biological functions in NPCs and cortical neurons differentiated from iPSCs derived from a KAT6A patient and healthy controls.

**Figure 6:**
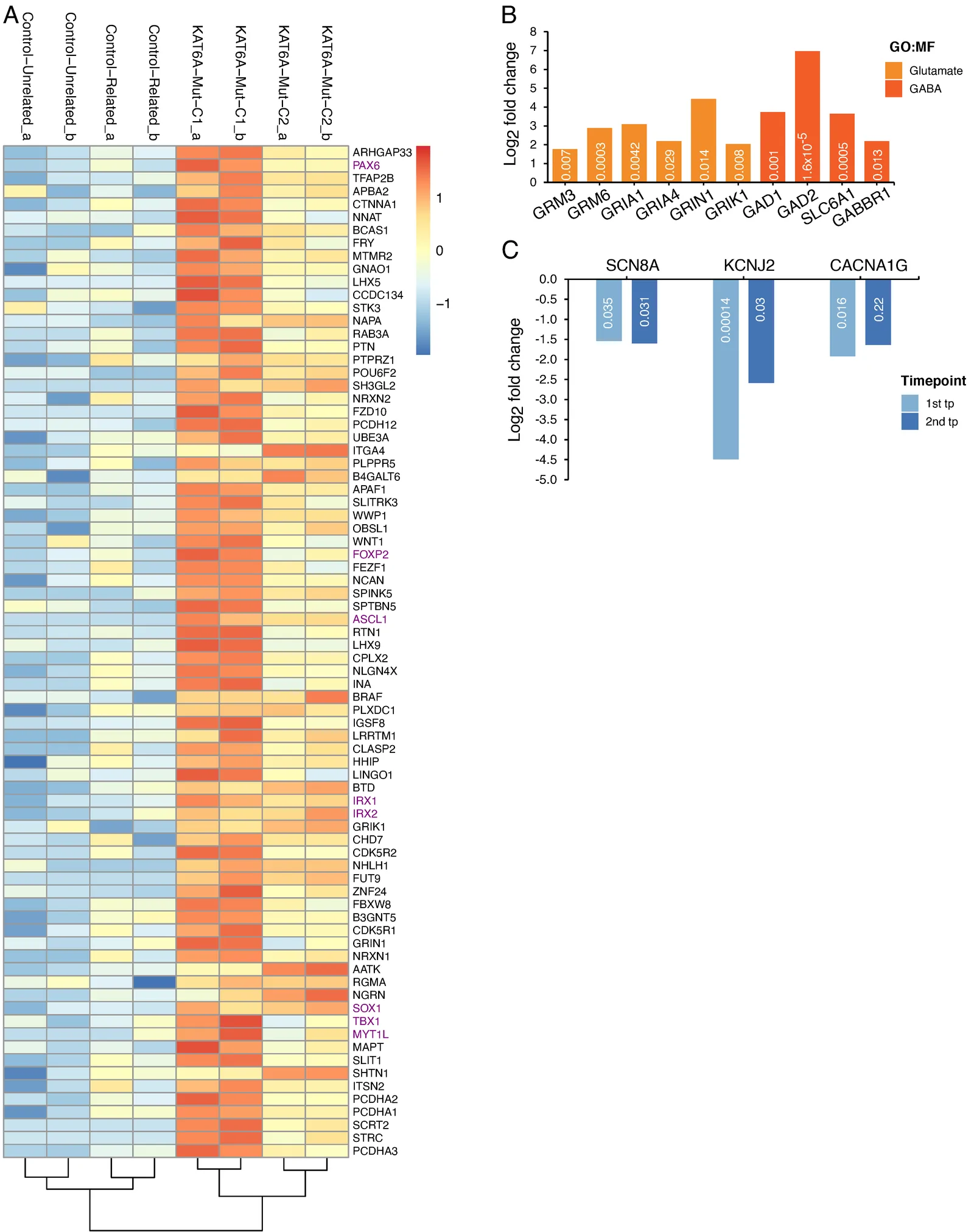
** Aberrant gene expression in neurons differentiated from *KAT6A*-mutant NPCs.** A) Heatmap of upregulated GO-BP -Nervous system development dysregulated genes in *KAT6A*-mutant at the 1^st^ tp of differentiation (78 genes). Upregulated TFs are marked in purple. B) Glutamate and GABA-related genes are upregulated in *KAT6A*-mutant neurons at the 1^st^ tp of differentiation (*p*-adj values are indicated inside each bar). C) The sodium channel gene *SCN8A*, potassium channel gene *KCNJ2* and the calcium channel gene *CACNA1G* are downregulated in *KAT6A*-mutant neurons (*p*-adj values are indicated inside each bar).

**Figure 7:**
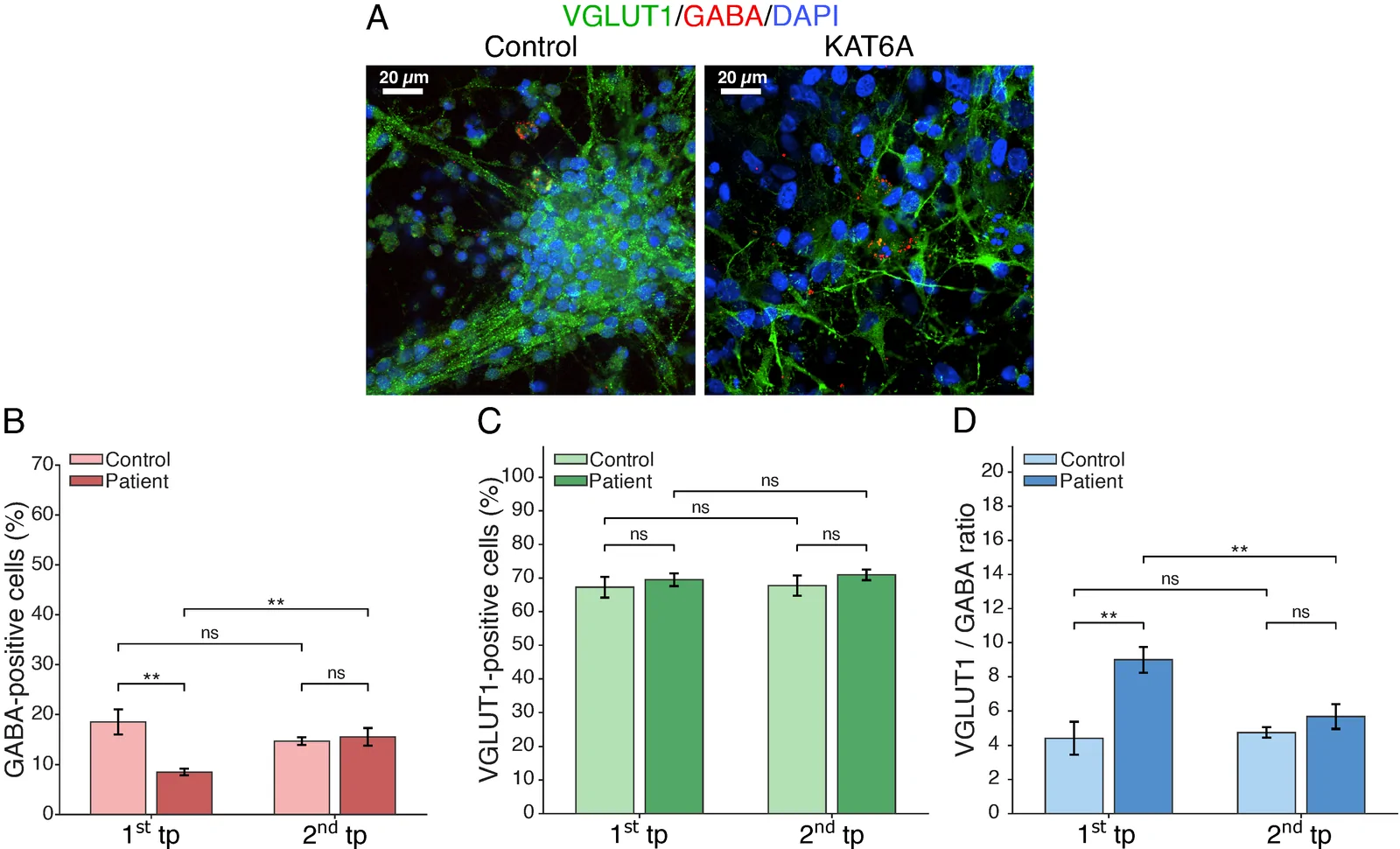
***KAT6A*-mutant neurons display a reduced percentage of GABA-positive neurons and an increased E/I ratio at the 1^st^ tp.** A) Representative images of immunofluorescence microscopy analysis of VGLUT1 (Green; a marker for glutamatergic neurons), GABA (red; a marker for inhibitory neurons) and DAPI (blue) at a 60X magnification for control and *KAT6A*-mutant iPSC-derived cortical neurons differentiated for 4-5 weeks. Scale bar denotes 20 µm. B) Quantification of immunofluorescence microscopy analysis for percentages of GABA-positive cells out of all cells (stained with DAPI) revealed significantly lower percentages in the *KAT6A*-mutant neurons at the 1^st^ tp compared to control neurons (*p* = 0.0046) and similar percentages at the 2^nd^ tp (*p* = 0.67). C) The percentages of VGLUT1-positive cells were similar between *KAT6A*-mutant neurons and control neurons in both time points (*p* = 0.55, *p* = 0.36, respectively). D) E/I ratio in *KAT6A*-mutant neuron culture is higher compared with the control in the 1^st^ tp and similar in the 2^nd^ tp (*p* = 0.0017, *p* = 0.25, respectively). Data are presented as mean ± SEM. ns, not significant.

**Figure 8:**
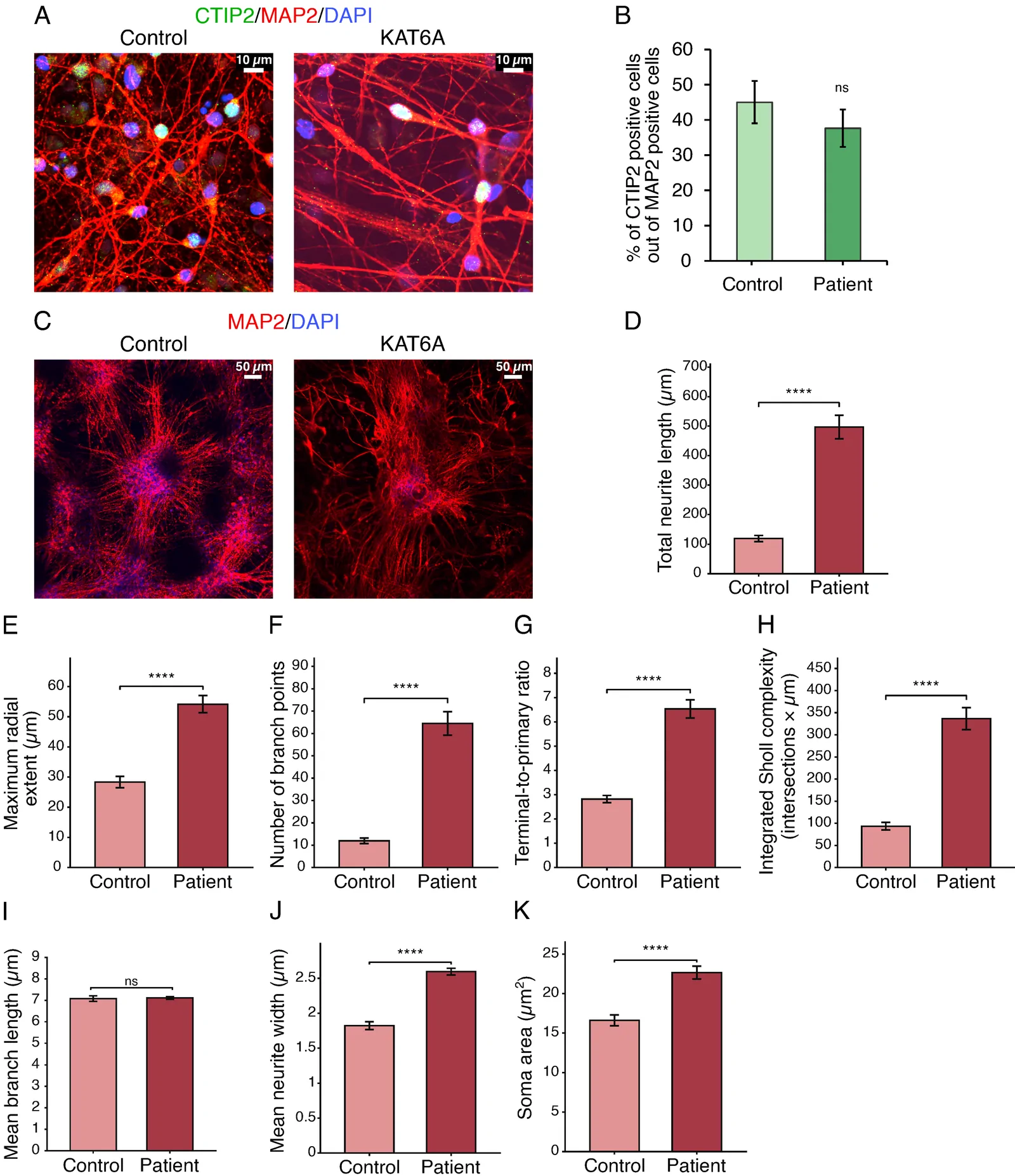
** Characterization of the morphology of iPSC-derived *KAT6A*-mutant cortical neurons.** A) A representative image of immunofluorescence analysis of MAP2 (red; a neuronal marker of dendrites and soma), CTIP2 (Green; a marker for cortical neurons), and DAPI (blue) at 60X for control and *KAT6A*-mutant iPSC-derived cortical neurons differentiated for 4-5 weeks. Scale bar denotes 10 µm. B) Quantification of immunofluorescence microscopy analysis in (A) for the percentage of CTIP2-positive cells out of MAP2-positive cells in control and *KAT6A*-mutant neurons. Data are presented as mean ± SD. C) Representative images of control and *KAT6A*-mutant neuronal networks following 4-5 weeks of differentiation that were immunostained for MAP2 and DAPI at 20X. Scale bar = 50 µm. D-I) Quantitative morphometric profiling of neuronal architecture revealed that, compared to control neurons, *KAT6A*-mutant neurons demonstrate elevated total neurite length (*p* = 3.2 x 10^-17^) (D), maximum radial extent (*p* = 3.2 x 10^-13^) (E), number of branch points (*p* = 6.6 x 10^-19^) (F), terminal to primary ratio (*p* = 7.52 x 10^-18^) (G), and integrated Sholl complexity (*p* = 7.9 x 10^-18^) (H). Mean branch length was similar between *KAT6A*-mutant neurons and control neurons (*p* = 0.83) (I). Mean neurite width (*p* = 7.67 x 10^-23^) (J) and soma size (*p* = 2.20 x 10^-08^) (K) were larger in *KAT6A*-mutant neurons compared with control neurons. Data are presented as mean ± SEM. ns, not significant.

**Figure 9:**
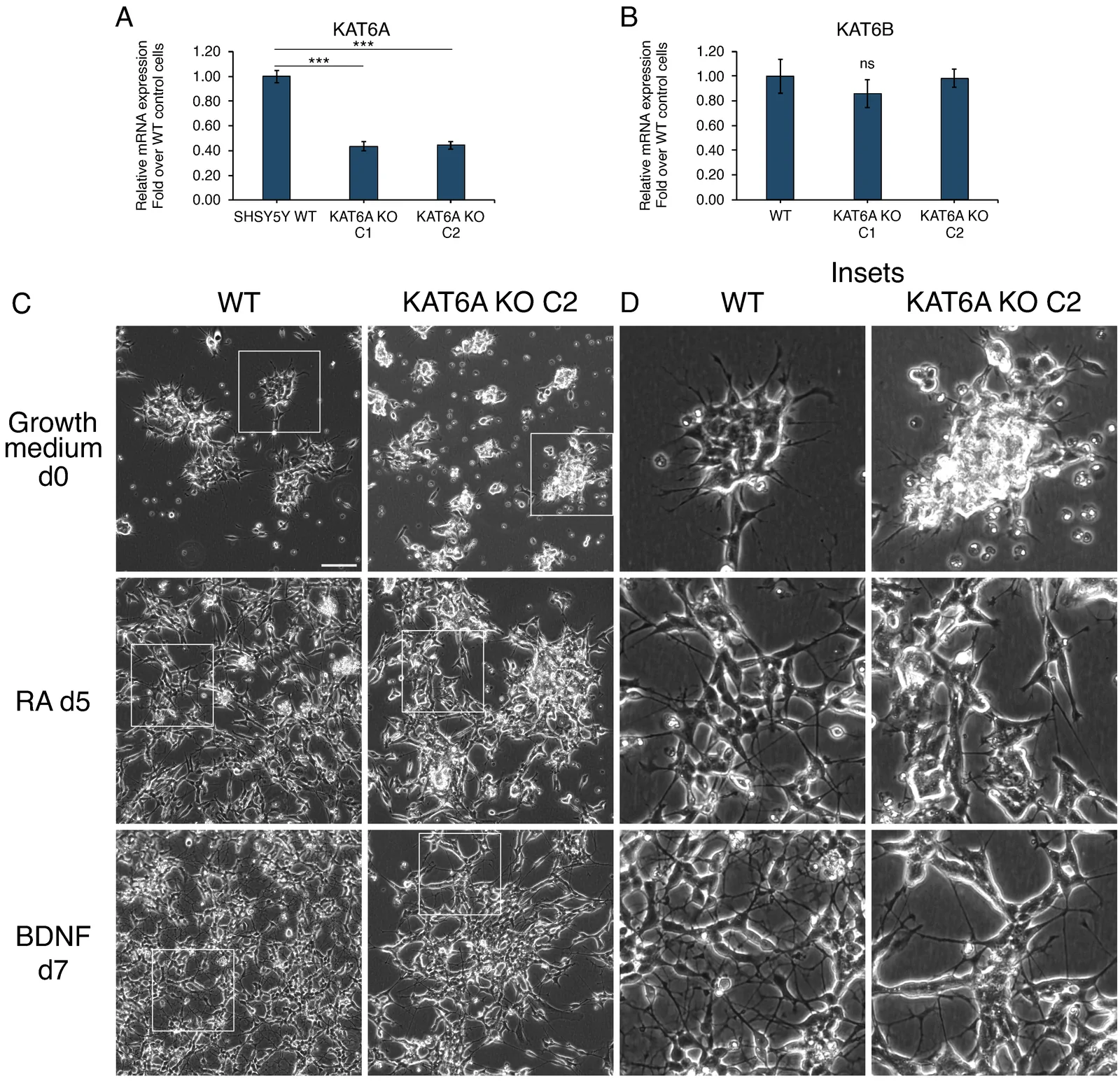
***KAT6A* KO SH-SY5Y cells display an attenuated differentiation upon RA and BDNF treatment.** A) RT-PCR shows ~60% reduction in mRNA levels of *KAT6A*. B) Relative gene expression of *KAT6B* in *KAT6A* KO SH-SY5Y cells. ns, not significant. The results represent mean ± SD of at least three independent experiments. C, D) KO of *KAT6A* attenuates neuronal differentiation of SH-SY5Y cells treated with RA and BDNF. Phase contrast microscopy images (at 20X magnification) of undifferentiated WT and *KAT6A* KO SH-SY5Y cells cultured in complete growth medium; cells treated with 10 µM RA for 5 days and cells treated with RA for 5 days followed by 50 ng/ml BDNF treatment for 2 days. Scale bar denotes 50 µm. D) Enlarged insets from microscope images appear in panel (C). The images are representative of the results obtained from three independent experiments.

**Figure 10:**
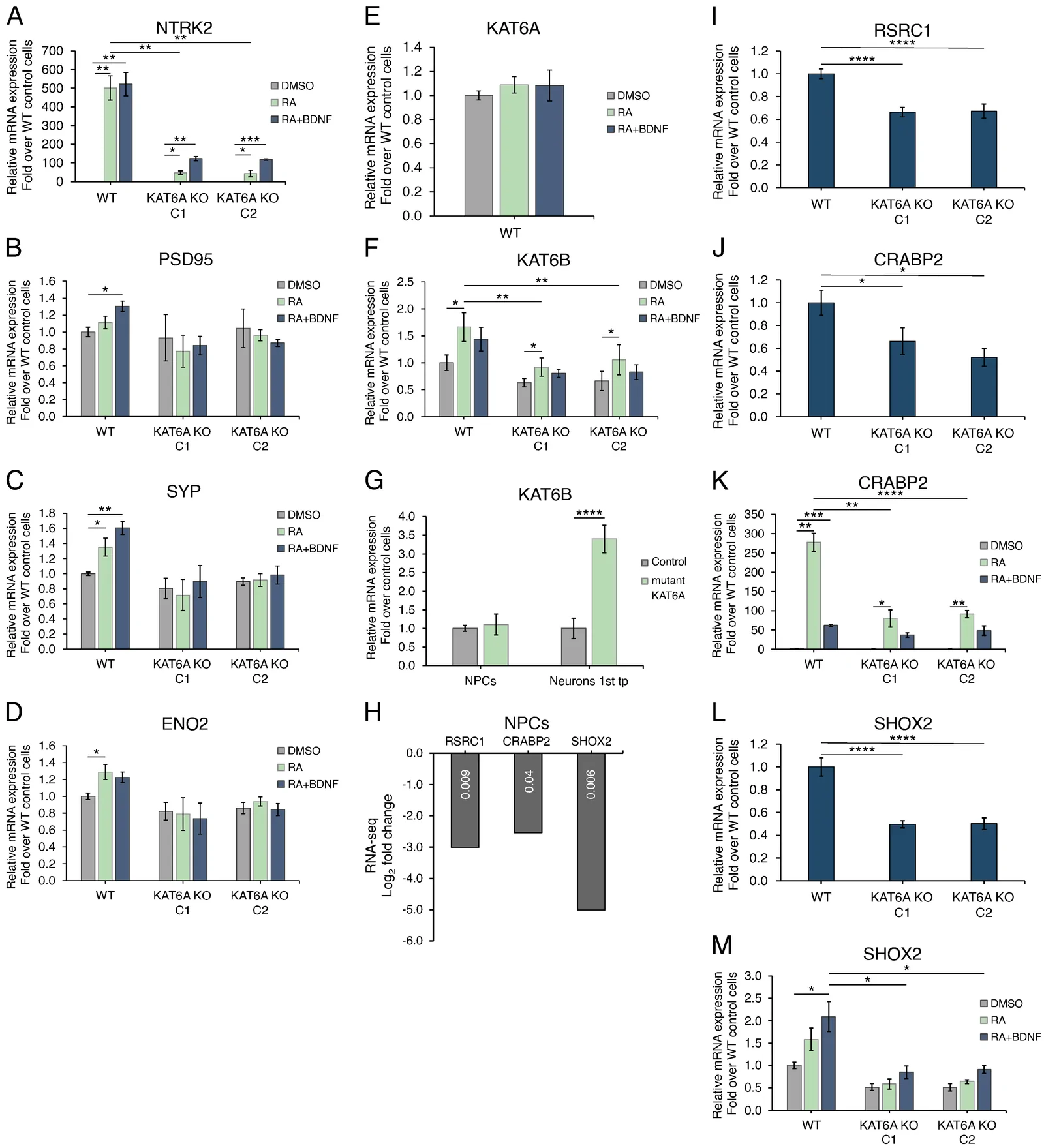
** Altered gene expression in *KAT6A* KO SH-SY5Y cells.** A-D) Relative gene expression of neuronal differentiation markers (*NTRK2*, *SYP*, *PSD95* and *ENO2*) in WT and *KAT6A* KO SH-SY5Y cells treated with DMSO/10 µM RA for 5 days and cells treated with RA for 5 days followed by 50 ng/ml BDNF treatment for 2 days. E, F) Relative gene expression of *KAT6A* (E) in WT cells and *KAT6B* (F) in WT and *KAT6A* KO SH-SY5Y cells following differentiation. G) *KAT6B* gene expression in *KAT6A*-mutant NPCs and young neurons compared with control cells. H) *RSRC1*, *CRABP2* and *SHOX2* gene expression is downregulated in RNA-seq of *KAT6A*-mutant NPCs (*p*-adj values are indicated inside each bar). I, J) *RSRC1* (I) and *CRABP2* (J) gene expression is also downregulated in *KAT6A* KO SH-SY5Y cells compared with WT cells. (K) Relative gene expression of *CRABP2* in WT and *KAT6A* KO SH-SY5Y cells following differentiation. L) *SHOX2* gene expression is downregulated in *KAT6A* KO SH-SY5Y cells compared with WT cells. M) Relative gene expression of *SHOX2* in WT and *KAT6A* KO SH-SY5Y cells following differentiation. The results represent mean ± SD of at least three independent experiments.

**Figure 11:**
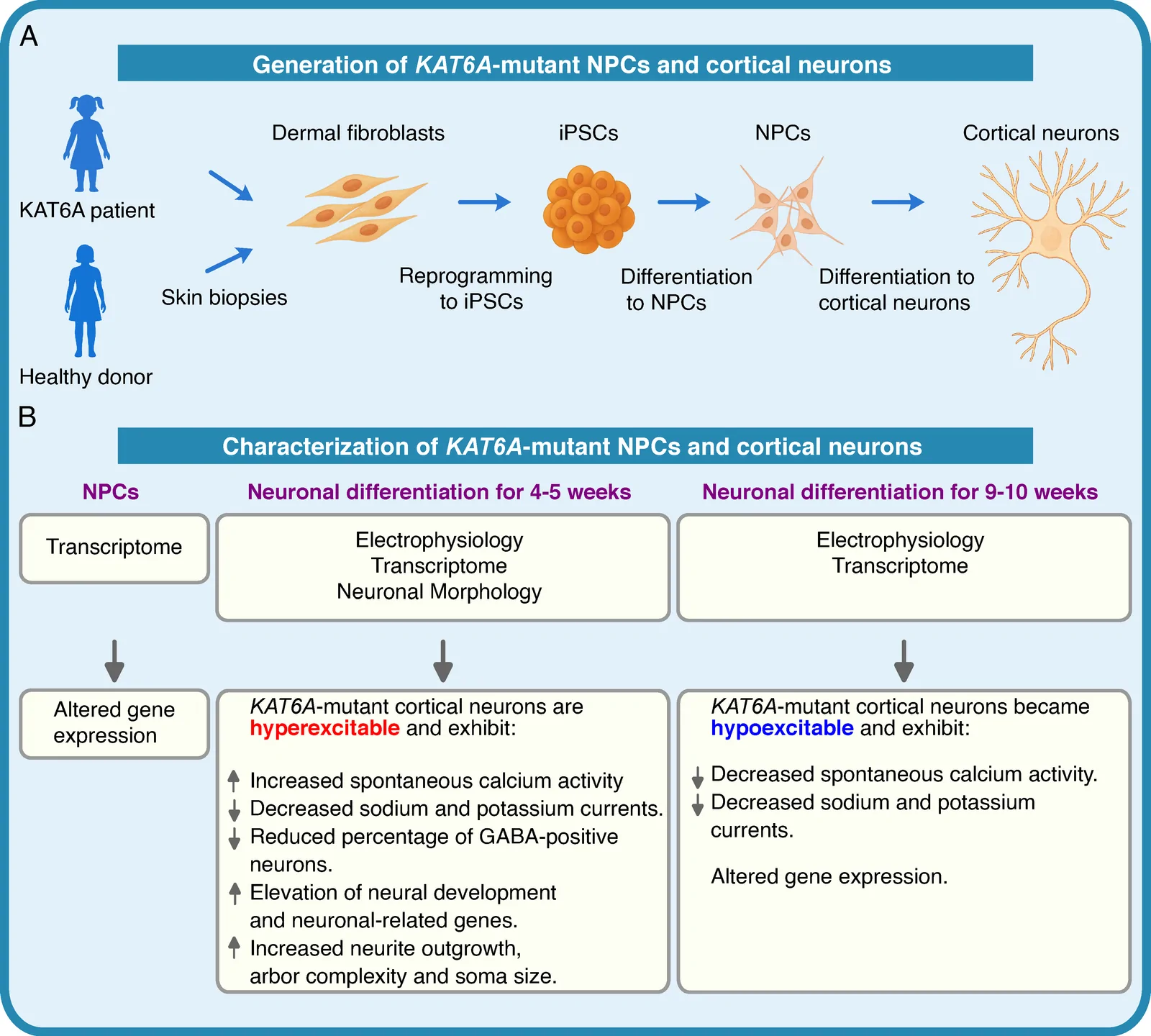
** Generation and characterization of NPCs and cortical neurons harboring a mutation in *KAT6A*.** A) Schematic diagram showing the generation of iPSC-derived NPCs and cortical neurons from a KAT6A syndrome patient and healthy individuals. B) Characterization of *KAT6A*-mutant NPCs and cortical neurons. *KAT6A*-mutant cortical neurons differentiated for 4-5 weeks displayed hyperexcitability and reduced sodium and potassium currents. Analysis of young *KAT6A*-mutant neurons revealed premature maturation that was characterized by the elevation of neural development and neuronal-related genes and increased total neurite length, neurite arborization, neurite width and soma size. Following 9-10 weeks of differentiation, *KAT6A*-mutant neurons exhibited hypoexcitability and reduced sodium and potassium currents.

## Data Availability

The data that supports the findings of this study are available in the supplementary material of this article or available from the corresponding author on reasonable request.

## Abbreviations

1^st^ tp: first time point
2^nd^ tp: second time point
AML: acute myeloid leukemia
APs: action potentials
ASD: autism spectrum disorder
BDNF: brain-derived neurotrophic factor
BP: biological process
CFC: Cardiofaciocutaneous
CNS: central nervous system
DEGs: differentially expressed genes
EBs: embryoid bodies
E/I: excitatory/inhibitory
FDR: false discovery rate
GABA: gamma-aminobutyric acid
GO: gene ontology
ID: intellectual disability
iPSCs: induced pluripotent stem cells
KO: knockout
LoF: loss-of-function
MF: molecular function
MRI: magnetic resonance imaging
NPCs: neural progenitor cells
RA: retinoic acid
RMP: Resting membrane potential
RSTS: Rubinstein-Taybi syndrome
sEPSCs: spontaneous excitatory postsynaptic currents
SFARI: Simons foundation autism research initiative
TF: transcription factor
VGLUT1: Vesicular glutamate transporter 1
WT: wild type

## References

[1] Champagne N, Pelletier N, Yang X-J (2001). The monocytic leukemia zinc finger protein MOZ is a histone acetyltransferase. Oncogene.

[2] Mousavi N, Yang XJ (2025). Lysine Acetyltransferase 6 Complexes in Neurodevelopmental Disorders and Different Types of Cancer. In: Halasa, M, Wawruszak, A. (eds) Histone and Non-Histone Reversible Acetylation in Development, Aging and Disease. Results and Problems in Cell Differentiation, vol. 75, Springer, Cham.

[3] Blasl AT, Schulze S, Qin C, Graf LG, Vogt R, Lammers M (2022). Post-translational lysine ac(et)ylation in health, ageing and disease. Biol Chem.

[4] Wiesel-Motiuk N, Assaraf YG (2020). The key roles of the lysine acetyltransferases KAT6A and KAT6B in physiology and pathology. Drug Resist Updat.

[5] Tan Y, Wang J, Ma F (2025). Lysine Acetyltransferase 6 in Health and Disease. MedComm.

[6] Al Ojaimi M, Banimortada BJ, Alragheb A, Hajir RS, Alves C, Walid D (2025). Molecular and clinical aspects of histone-related disorders. Hum Genomics.

[7] Ng R, Kalinousky A, Harris J (2023). Epigenetics of cognition and behavior: insights from Mendelian disorders of epigenetic machinery. J Neurodev Disord.

[8] Park J, Lee K, Kim K, Yi SJ (2022). The role of histone modifications: from neurodevelopment to neurodiseases. Signal Transduct Target Ther.

[9] Borrow J, Stanton VP, Andresen JM, Becher R, Behm FG, Chaganti RSK (1996). The translocation t(8;16)(p11;p13) of acute myeloid leukaemia fuses a putative acetyltransferase to the CREB-binding protein. Nat Genet.

[10] Katsumoto T, Yoshida N, Kitabayashi I (2008). Roles of the histone acetyltransferase monocytic leukemia zinc finger protein in normal and malignant hematopoiesis. Cancer Sci.

[11] Tham E, Lindstrand A, Santani A, Malmgren H, Nesbitt A, Dubbs HA (2015). Dominant mutations in KAT6A cause intellectual disability with recognizable syndromic features. Am J Hum Genet.

[12] Arboleda VA, Lee H, Dorrani N, Zadeh N, Willis M, Macmurdo CF (2015). De novo nonsense mutations in KAT6A, a lysine acetyl-transferase gene, cause a syndrome including microcephaly and global developmental delay. Am J Hum Genet.

[13] Millan F, Cho MT, Retterer K, Monaghan KG, Bai R, Vitazka P (2016). Whole exome sequencing reveals de novo pathogenic variants in KAT6A as a cause of a neurodevelopmental disorder. Am J Med Genet Part A.

[14] Kennedy J, Goudie D, Blair E, Chandler K, Joss S, McKay V (2018). KAT6A Syndrome: genotype-phenotype correlation in 76 patients with pathogenic KAT6A variants. Genet Med.

[15] Smith C, Harris J (2021). Sleep, behavior, and adaptive function in KAT6A syndrome. Brain Sci.

[16] Urreizti R, Lopez-Martin E, Martinez-Monseny A, Pujadas M, Castilla-Vallmanya L, Pérez-Jurado LA (2020). Five new cases of syndromic intellectual disability due to KAT6A mutations: widening the molecular and clinical spectrum. Orphanet J Rare Dis.

[17] St John M, Amor DJ, Morgan AT (2022). Speech and language development and genotype-phenotype correlation in 49 individuals with KAT6A syndrome. Am J Med Genet Part A.

[18] Ng R, Kalinousky AJ, Harris J (2024). Neuropsychological profile associated with KAT6A syndrome: Emergent genotype-phenotype trends. Orphanet J Rare Dis.

[19] Voss AK, Collin C, Dixon MP, Thomas T (2009). Moz and Retinoic Acid Coordinately Regulate H3K9 Acetylation, Hox Gene Expression, and Segment Identity. Dev Cell.

[20] Katsumoto T, Aikawa Y, Iwama A, Ueda S, Ichikawa H, Ochiya T (2006). MOZ is essential for maintenance of hematopoietic stem cells. Genes Dev.

[21] Thomas T, Corcoran LM, Gugasyan R, Dixon MP, Brodnicki T, Nutt SL (2006). Monocytic leukemia zinc finger protein is essential for the development of long-term reconstituting hematopoietic stem cells. Genes Dev.

[22] Miller CT, Maves L, Kimmel CB (2004). moz regulates Hox expression and pharyngeal segmental identity in zebrafish. Development.

[23] Crump JG, Swartz ME, Eberhart JK, Kimmel CB (2006). Moz-dependent Hox expression controls segment-specific fate maps of skeletal precursors in the face. Development.

[24] Vanyai HK, Garnham A, May RE, McRae HM, Collin C, Wilcox S (2019). MOZ directs the distal-less homeobox gene expression program during craniofacial development. Development.

[25] Liu Y, Fan M, Yang J, Mihaljević L, Chen KH, Ye Y (2024). KAT6A deficiency impairs cognitive functions through suppressing RSPO2/Wnt signaling in hippocampal CA3. Sci Adv.

[26] Eccles S, Vanyai HK, Bergamasco MI, Malelang S, Pehlivanoglu H, Garnham AL (2026). Acetyl-carnitine improves hyperactivity and learning deficits in KAT6A haploinsufficient mice. Life Sci Alliance.

[27] Romanovsky E, Choudhary A, Peles D, Abu-Akel A, Stern S (2025). Uncovering convergence and divergence between autism and schizophrenia using genomic tools and patients' neurons. Mol Psychiatry.

[28] Schafer ST, Paquola ACM, Stern S, Gosselin D, Ku M, Pena M (2019). Pathological priming causes developmental gene network heterochronicity in autistic subject-derived neurons. Nat Neurosci.

[29] Hussein Y, Tripathi U, Choudhary A, Nayak R, Peles D, Rosh I (2023). Early maturation and hyperexcitability is a shared phenotype of cortical neurons derived from different ASD-associated mutations. Transl Psychiatry.

[30] Brant B, Stern T, Shekhidem HA, Mizrahi L, Rosh I, Stern Y (2021). IQSEC2 mutation associated with epilepsy, intellectual disability, and autism results in hyperexcitability of patient-derived neurons and deficient synaptic transmission. Mol Psychiatry.

[31] Nayak R, Rosh I, Rabinski T, Falik D, Mendel Percia M, Stern S (2022). Generation and characterization of iPSC lines (UOHi003-A, UOHi002-A) from a patient with SHANK3 mutation and her healthy mother. Stem Cell Res.

[32] Brennand KJ, Simone A, Jou J, Gelboin-Burkhart C, Tran N, Sangar S (2011). Modelling schizophrenia using human induced pluripotent stem cells. Nature.

[33] Rosh I, Tripathi U, Hussein Y, Rike WA, Djamus J, Shklyar B (2024). Synaptic dysfunction and extracellular matrix dysregulation in dopaminergic neurons from sporadic and E326K-GBA1 Parkinson's disease patients. Npj Park Dis.

[34] Otsu N (1979). A Threshold Selection Method from Gray-Level Histograms. IEEE Trans Syst Man Cybern.

[35] Soille P (1991). Spatial distributions from contour lines: An efficient methodology based on distance transformations. J Vis Commun Image Represent.

[36] Sholl DA (1953). Dendritic organization in the neurons of the visual and motor cortices of the cat. J Anat.

[37] Hager NA, McAtee CK, Lesko MA, O'Donnell AF (2022). Inwardly Rectifying Potassium Channel Kir2.1 and its “Kir-ious” Regulation by Protein Trafficking and Roles in Development and Disease. Front Cell Dev Biol.

[38] Lv D, Jia F, Hou Y, Sang Y, Alvarez AA, Zhang W (2017). Histone acetyltransferase KAT6A upregulates PI3K/AKT signaling through TRIM24 binding. Cancer Res.

[39] Xie W, Zhang Y, Wang B, Hu Y, Zhan B, Wei F (2020). Tripartite motif containing 24 regulates cell proliferation in colorectal cancer through YAP signaling. Cancer Med.

[40] Tan K, Wang J, Su X, Zheng Y, Li W (2024). KAT6A/YAP/TEAD4 pathway modulates osteoclastogenesis by regulating the RANKL/OPG ratio on the compression side during orthodontic tooth movement. Prog Orthod.

[41] Kikkawa T, Casingal CR, Chun SH, Shinohara H, Hiraoka K, Osumi N (2019). The role of Pax6 in brain development and its impact on pathogenesis of autism spectrum disorder. Brain Res.

[42] Mall M, Kareta MS, Chanda S, Ahlenius H, Perotti N, Zhou B (2017). Myt1l safeguards neuronal identity by actively repressing many non-neuronal fates. Nature.

[43] Co M, Anderson AG, Konopka G (2020). FOXP transcription factors in vertebrate brain development, function, and disorders. Wiley Interdiscip Rev Dev Biol.

[44] Kan L, Israsena N, Zhang Z, Hu M, Zhao LR, Jalali A (2004). Sox1 acts through multiple independent pathways to promote neurogenesis. Dev Biol.

[45] Catela C, Assimacopoulos S, Chen Y, Tsioras K, Feng W, Kratsios P (2025). The Iroquois (Iro/Irx) homeobox genes are conserved Hox targets involved in motor neuron development. iScience.

[46] Liu Y, Schubert J, Sonnenberg L, Helbig KL, Hoei-Hansen CE, Koko M (2019). Neuronal mechanisms of mutations in SCN8A causing epilepsy or intellectual disability. Brain.

[47] Lory P, Nicole S, Monteil A (2020). Neuronal Cav3 channelopathies: recent progress and perspectives. Pflugers Arch Eur J Physiol.

[48] Xiao J, Cheng X, Huang D, Wei S, Hu Z (2025). Functions of the KCNE Gene Family in Ion Channels. Biochem Genet.

[49] Targett IL, Crompton LA, Conway ME, Craig TJ (2024). Differentiation of SH-SY5Y neuroblastoma cells using retinoic acid and BDNF: a model for neuronal and synaptic differentiation in neurodegeneration. Vitr Cell Dev Biol - Anim.

[50] Encinas M, Iglesias M, Liu Y, Wang H, Muhaisen A, Ceña V (2000). Sequential treatment of SH-SY5Y cells with retinoic acid and brain-derived neurotrophic factor gives rise to fully differentiated, neurotrophic factor-dependent, human neuron-like cells. J Neurochem.

[51] Goldie BJ, Barnett MM, Cairns MJ (2014). BDNF and the maturation of posttranscriptional regulatory networks in human SH-SY5Y neuroblast differentiation. Front Cell Neurosci.

[52] Kovalevich J, Langford D (2013). Considerations for the use of SH-SY5Y neuroblastoma cells in neurobiology. Methods Mol Biol.

[53] Thomson AC, Schuhmann T, de Graaf TA, Sack AT, Rutten BPF, Kenis G (2022). The Effects of Serum Removal on Gene Expression and Morphological Plasticity Markers in Differentiated SH-SY5Y Cells. Cell Mol Neurobiol.

[54] Murillo JR, Goto-Silva L, Sánchez A, Nogueira FCS, Domont GB, Junqueira M (2017). Quantitative proteomic analysis identifies proteins and pathways related to neuronal development in differentiated SH-SY5Y neuroblastoma cells. EuPA Open Proteomics.

[55] Perez Y, Menascu S, Cohen I, Kadir R, Basha O, Shorer Z (2018). RSRC1 mutation affects intellect and behaviour through aberrant splicing and transcription, downregulating IGFBP3. Brain.

[56] Oppenheimer O, Cheung NK, Gerald WL (2007). The RET oncogene is a critical component of transcriptional programs associated with retinoic acid-induced differentiation in neuroblastoma. Mol Cancer Ther.

[57] Gu SC, Shen CY, Deng JQ, Zhang W, Zeng SL, Hao Y (2025). The human cerebral cortex morphology in neuropsychiatric disorders: A causal inference based on Mendelian Randomization. J Affect Disord.

[58] Chini M, Hanganu-Opatz IL (2021). Prefrontal Cortex Development in Health and Disease: Lessons from Rodents and Humans. Trends Neurosci.

[59] Cho YT, Fudge JL, Ross DA (2016). The Architecture of Cortex-in Illness and in Health. Biol Psychiatry.

[60] Quraishi IH, Stern S, Mangan KP, Zhang Y, Ali SR, Mercier MR (2019). An epilepsy-associated KCNT1 mutation enhances excitability of human iPSC-derived neurons by increasing slack KNa currents. J Neurosci.

[61] Meisler MH, Hill SF, Yu W (2021). Sodium channelopathies in neurodevelopmental disorders. Nat Rev Neurosci.

[62] Wagnon JL, Barker BS, Ottolini M, Park Y, Volkheimer A, Valdez P (2017). Loss-of-function variants of SCN8A in intellectual disability without seizures. Neurol Genet.

[63] Johannesen KM, Liu Y, Koko M, Gjerulfsen CE, Sonnenberg L, Schubert J (2022). Genotype-phenotype correlations in SCN8A-related disorders reveal prognostic and therapeutic implications. Brain.

[64] Boulis NM, Handy CR, Krudy CA, Donnelly EM, Federici T, Franz CK (2013). Regulated neuronal neuromodulation via spinal cord expression of the gene for the inwardly rectifying potassium channel 2.1 (Kir2.1). Neurosurgery.

[65] Ma C, Rosenzweig J, Zhang P, Johns DC, LaMotte RH (2010). Expression of inwardly rectifying potassium channels by an inducible adenoviral vector reduced the neuronal hyperexcitability and hyperalgesia produced by chronic compression of the spinal ganglion. Mol Pain.

[66] Johns DC, Marx R, Mains RE, O'Rourke B, Marbán E (1999). Inducible genetic suppression of neuronal excitability. J Neurosci.

[67] Xue M, Atallah B V, Scanziani M (2014). Equalizing excitation-inhibition ratios across visual cortical neurons. Nature.

[68] Li K, Savitska D, Garaschuk O (2024). K+ channel-mediated retarded maturation of interneurons and its role in neurodevelopmental disorders. Neural Regen Res.

[69] Binda A, Rivolta I, Villa C, Chisci E, Beghi M, Cornaggia CM (2018). A novel KCNJ2 mutation identified in an autistic proband affects the single channel properties of Kir2.1. Front Cell Neurosci.

[70] Essa MM, Braidy N, Subash S, Vijayan RK, Guillemin GJ (2022). Excitotoxicity in the Pathogenesis of Autism. In: Kostrzewa, R.M. (eds) Handbook of Neurotoxicity. Springer, Cham. vol. 3.

[71] Nisar S, Bhat AA, Masoodi T, Hashem S, Akhtar S, Ali TA (2022). Genetics of glutamate and its receptors in autism spectrum disorder. Mol Psychiatry.

[72] Verma M, Lizama BN, Chu CT (2022). Excitotoxicity, calcium and mitochondria: a triad in synaptic neurodegeneration. Transl Neurodegener.

[73] Ben Mahmoud M, Rátkai A, Bauer K, Bencsik N, Szücs A, Schlett K (2025). Multifactorial approach is needed to unravel the maturation phases of human neurons derived from induced pluripotent stem cells. Sci Reports.

[74] Nagy J, Kobolák J, Berzsenyi S, Ábrahám Z, Avci HX, Bock I (2017). Altered neurite morphology and cholinergic function of induced pluripotent stem cell-derived neurons from a patient with Kleefstra syndrome and autism. Transl Psychiatry.

[75] Quach TT, Stratton HJ, Khanna R, Kolattukudy PE, Honnorat J, Meyer K (2021). Intellectual disability: dendritic anomalies and emerging genetic perspectives. Acta Neuropathol.

[76] Granato A, Merighi A (2022). Dendrites of Neocortical Pyramidal Neurons: The Key to Understand Intellectual Disability. Cell Mol Neurobiol.

[77] Goikolea-Vives A, Stolp HB (2021). Connecting the neurobiology of developmental brain injury: Neuronal arborisation as a regulator of dysfunction and potential therapeutic target. Int J Mol Sci.

[78] Alari V, Russo S, Terragni B, Ajmone PF, Sironi A, Catusi I (2018). iPSC-derived neurons of CREBBP- and EP300-mutated Rubinstein-Taybi syndrome patients show morphological alterations and hypoexcitability. Stem Cell Res.

[79] Yeh E, Dao DQ, Wu ZY, Kandalam SM, Camacho FM, Tom C (2018). Patient-derived iPSCs show premature neural differentiation and neuron type-specific phenotypes relevant to neurodevelopment. Mol Psychiatry.

[80] Hebron KE, Hernandez ER, Yohe ME (2022). The RASopathies: from pathogenetics to therapeutics. Dis Model Mech.

[81] Tidyman WE, Rauen KA (2009). The RASopathies: developmental syndromes of Ras/MAPK pathway dysregulation. Curr Opin Genet Dev.

[82] Ciceri G, Baggiolini A, Cho HS, Kshirsagar M, Benito-Kwiecinski S, Walsh RM (2024). An epigenetic barrier sets the timing of human neuronal maturation. Nature.

[83] Qiao Y, Wang R, Yang X, Tang K, Jing N (2014). Dual Roles of Histone H3 Lysine 9 Acetylation in Human Embryonic Stem Cell Pluripotency and Neural Differentiation. J Biol Chem.

[84] Nothof SA, Magdinier F, Van-Gils J (2022). Chromatin Structure and Dynamics: Focus on Neuronal Differentiation and Pathological Implication. Genes (Basel).

[85] Bergamasco MI, Abeysekera W, Garnham AL, Hu Y, Li-Wai-suen CSN, Sheikh BN (2024). KAT6B is required for histone 3 lysine 9 acetylation and SOX gene expression in the developing brain. Life Sci Alliance.

[86] Fu JY, Huang SJ, Wang BL, Yin JH, Chen CY, Xu JB (2024). Lysine acetyltransferase 6A maintains CD4+ T cell response via epigenetic reprogramming of glucose metabolism in autoimmunity. Cell Metab.

[87] Bergamasco MI, Ozturk E, Casillas-Espinosa PM, Garnham AL, Abeysekera W, Wimmer VC (2025). KAT6B overexpression in mice causes aggression, anxiety, and epilepsy. iScience.

[88] Bergamasco MI, Vanyai HK, Garnham AL, Geoghegan ND, Vogel AP, Eccles S (2024). Increasing histone acetylation improves sociability and restores learning and memory in KAT6B-haploinsufficient mice. J Clin Invest.

[89] Voss AK, Vanyai HK, Collin C, Dixon MP, McLennan TJ, Sheikh BN (2012). MOZ Regulates the Tbx1 Locus, and Moz Mutation Partially Phenocopies DiGeorge Syndrome. Dev Cell.

[90] Newman DM, Sakaguchi S, Lun A, Preston S, Pellegrini M, Khamina K (2016). Acetylation of the Cd8 Locus by KAT6A Determines Memory T Cell Diversity. Cell Rep.

[91] Thomas T, Voss AK, Chowdhury K, Gruss P (2000). Querkopf, a MYST family histone acetyltransferase, is required for normal cerebral cortex development. Development.

[92] Merson TD, Dixon MP, Collin C, Rietze RL, Bartlett PF, Thomas T (2006). The transcriptional coactivator Querkopf controls adult neurogenesis. J Neurosci.

[93] Santos-Terra J, Deckmann I, Fontes-Dutra M, Schwingel GB, Bambini-Junior V, Gottfried C (2021). Transcription factors in neurodevelopmental and associated psychiatric disorders: A potential convergence for genetic and environmental risk factors. Int J Dev Neurosci.

[94] Stevanovic M, Drakulic D, Lazic A, Ninkovic DS, Schwirtlich M, Mojsin M (2021). SOX Transcription Factors as Important Regulators of Neuronal and Glial Differentiation During Nervous System Development and Adult Neurogenesis. Front Mol Neurosci.

[95] Manuel MN, Mi D, Masonand JO, Price DJ (2015). Regulation of cerebral cortical neurogenesis by the Pax6 transcription factor. Front Cell Neurosci.

[96] Thakurela S, Tiwari N, Schick S, Garding A, Ivanek R, Berninger B (2016). Mapping gene regulatory circuitry of Pax6 during neurogenesis. Cell Discov.

[97] Kim KC, Lee DK, Go HS, Kim P, Choi CS, Kim JW (2014). Pax6-dependent cortical glutamatergic neuronal differentiation regulates autism-like behavior in prenatally valproic acid-exposed rat offspring. Mol Neurobiol.

[98] Osumi N, Shinohara H, Numayama-Tsuruta K, Maekawa M (2008). Concise Review: Pax6 Transcription Factor Contributes to both Embryonic and Adult Neurogenesis as a Multifunctional Regulator. Stem Cells.

[99] Tsui D, Vessey JP, Tomita H, Kaplan DR, Miller FD (2013). FoxP2 regulates neurogenesis during embryonic cortical development. J Neurosci.

[100] Spiteri E, Konopka G, Coppola G, Bomar J, Oldham M, Ou J (2007). Identification of the Transcriptional Targets of FOXP2, a Gene Linked to Speech and Language, in Developing Human Brain. Am J Hum Genet.

[101] Flore G, Cioffi S, Bilio M, Illingworth E (2017). Cortical Development Requires Mesodermal Expression of Tbx1, a Gene Haploinsufficient in 22q11.2 Deletion Syndrome. Cereb Cortex.

[102] Wang B, Vartak R, Hennick KM, Zaltsman Y, Naing ZZC, Polacco BJ (2026). Autism mutations rewire protein interaction networks to drive neurodevelopmental pathology. Science.

[103] Ghyselinck NB, Duester G (2019). Retinoic acid signaling pathways. Dev.

[104] Novitch BG, Wichterle H, Jessell TM, Sockanathan S (2003). A Requirement for Retinoic Acid-Mediated Transcriptional Activation in Ventral Neural Patterning and Motor Neuron Specification. Neuron.

[105] Koshy AM, Mendoza-Parra MA (2023). Retinoids: Mechanisms of Action in Neuronal Cell Fate Acquisition. Life.

[106] Siegenthaler JA, Ashique AM, Zarbalis K, Patterson KP, Hecht JH, Kane MA (2009). Retinoic acid from the meninges regulates cortical neuron generation. Cell.

[107] Yan F, Li J, Milosevic J, Petroni R, Liu S, Shi Z (2022). KAT6A and ENL Form an Epigenetic Transcriptional Control Module to Drive Critical Leukemogenic Gene-Expression Programs. Cancer Discov.

[108] Nava AA, Jops CT, Vuong CK, Niles-Jensen SL, Bondhus L, Ong CJ (2023). KAT6A mutations drive transcriptional dysregulation of cell cycle and Autism risk genes in an Arboleda-Tham Syndrome cerebral organoid model. BioRxiv.

[109] Xu J, Wang L, Li H, Yang T, Zhang Y, Hu T (2019). Shox2 regulates osteogenic differentiation and pattern formation during hard palate development in mice. J Biol Chem.

[110] Vickerman L, Neufeld S, Cobb J (2011). Shox2 function couples neural, muscular and skeletal development in the proximal forelimb. Dev Biol.

[111] Scott A, Hasegawa H, Sakurai K, Yaron A, Cobb J, Wang F (2011). Transcription Factor Short Stature Homeobox 2 Is Required for Proper Development of Tropomyosin-Related Kinase B-Expressing Mechanosensory Neurons. J Neurosci.

[112] Rosin JM, Kurrasch DM, Cobb J (2015). Shox2 is required for the proper development of the facial motor nucleus and the establishment of the facial nerves. BMC Neurosci.

[113] Hongbing L, Espinoza-Lewis RA, Chen C, Hu X, Zhang Y, Chen YP (2012). The role of Shox2 in SAN development and function. Pediatr. Cardiol.

[114] Yu Di, Febbo IG, Maroteaux MJ, Wang H, Song Y, Han X (2021). The Transcription Factor Shox2 Shapes Neuron Firing Properties and Suppresses Seizures by Regulation of Key Ion Channels in Thalamocortical Neurons. Cereb Cortex.

[115] Kunii M, Doi H, Hashiguchi S, Matsuishi T, Sakai Y, Iai M (2020). De novo CACNA1G variants in developmental delay and early-onset epileptic encephalopathies. J Neurol Sci.

[116] Kessi M, Chen B, Peng J, Yan F, Yang L, Yin F (2021). Calcium channelopathies and intellectual disability: a systematic review. Orphanet J Rare Dis.

[117] Qebibo L, Davakan A, Nesson-Dauphin M, Boulali N, Siquier-Pernet K, Afenjar A (2025). The characterization of new de novo CACNA1G variants affecting the intracellular gate of Cav3.1 channel broadens the spectrum of neurodevelopmental phenotypes in SCA42ND. Genet Med.

