# Supplementary material for: KAT6A-Mutant iPSC-Derived Cortical Neurons Display Precocious Maturation, Dysregulated Electrophysiology, and Altered Morphology
Source: Int J Biol Sci. 2026 Sep 24;22(15):8448–75. doi: 10.7150/ijbs.134210 (PMC13685762; doi:10.7150/ijbs.134210)
Supplement: Supplementary file 1 [file ijbsv22p8448s1.pdf]

Supplementary Material

KAT6A-Mutant iPSC-Derived Cortical Neurons Display Precocious Maturation, Dysregulated Electrophysiology, and Altered Morphology

Naama Wiesel-Motiuk[1#], Ashwani Choudhary[2#], Yara Hussein[2#], Utkarsh Tripathi[2], Wote Amelo Rike[2], Aviram Shemen[2], Ritu Nayak[2], Souvik Chakraborty[2],Omveer Sharma[2], Tatiana Rabinski[3,4,5], Michal Stark[1], Karin Weiss[6,7], Gad D. Vatine[3,4,5], Shani Stern[2*], and Yehuda G. Assaraf[1*]

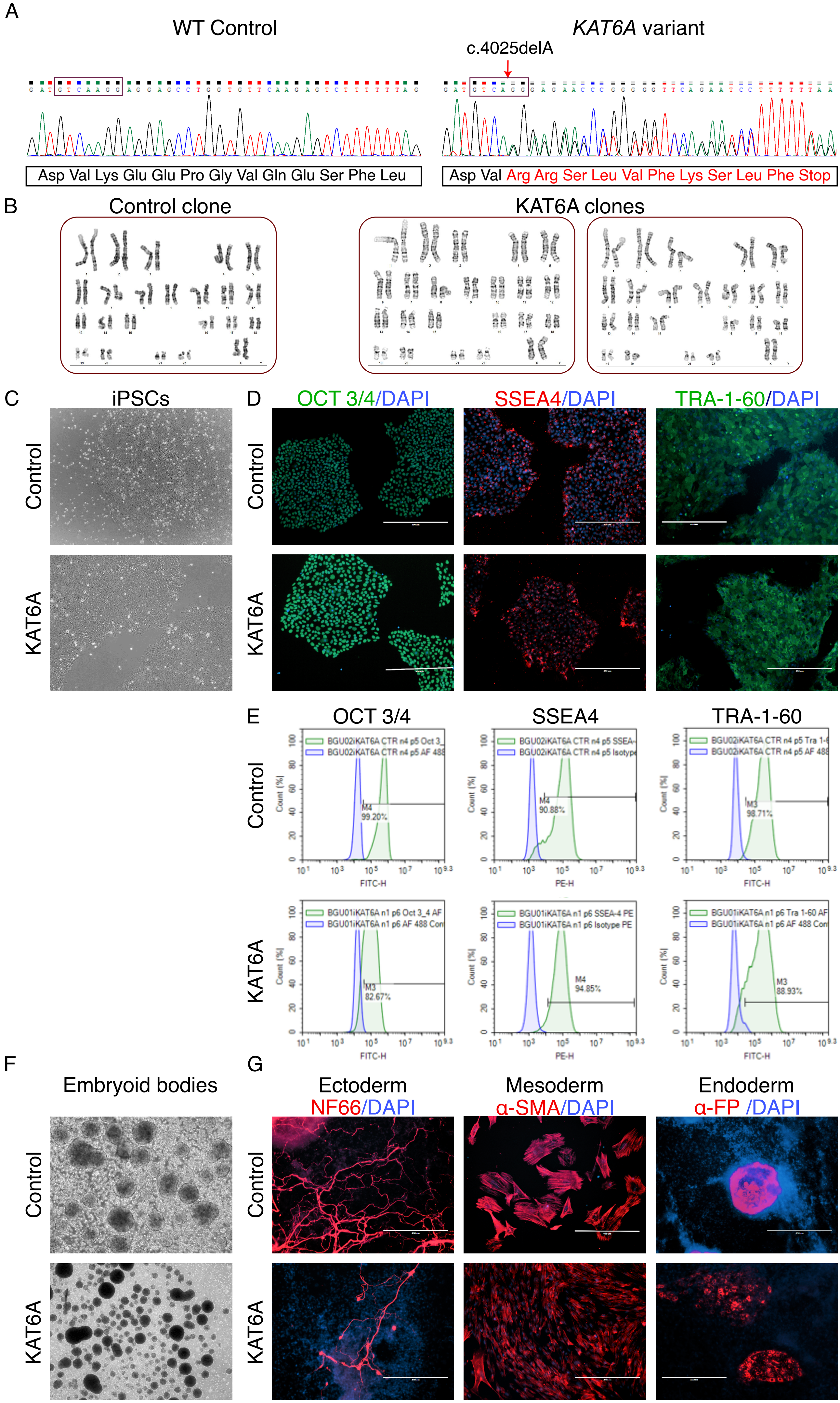

A
WT Control
KAT6A variant
c.4025delA
Asp Val Lys Glu Glu Pro Gly Val Gln Glu Ser Phe Leu
Asp Val Arg Arg Ser Leu Val Phe Lys Ser Leu Phe Stop
B
Control clone
KAT6A clones
C
iPSCs
Control
KAT6A
D
OCT 3/4/DAPI
SSEA4/DAPI
TRA-1-60/DAPI
Control
KAT6A
E
OCT 3/4
SSEA4
TRA-1-60
Control
KAT6A
M4 99.20%
M4 90.88%
M3 98.71%
M3 82.67%
M4 94.85%
M3 88.93%
Count [%]
FITC-H
PE-H
F
Embryoid bodies
Control
KAT6A
G
Ectoderm
NF66/DAPI
Mesoderm
α-SMA/DAPI
Endoderm
α-FP /DAPI
Control
KAT6A

Figure S1. Characterization of iPSC clones derived from a KAT6A syndrome patient and a related control. A) Sanger sequencing of a DNA fragment from exon 17 validated the retention of a heterozygous *KAT6A* c.4025delA pathogenic variant in the patient's iPSC cell line, compared to the sequence of the related unaffected control line. The location of the mutation and the altered amino acid sequence of ten missense mutations followed by a stop codon are indicated (framed by rectangles). B) iPSC clones of the related control and KAT6A syndrome patient maintained a normal 46,XX karyotype and showed no abnormalities. C) Images of iPSC colonies that were generated from dermal fibroblasts derived from the patient and the related unaffected control. (D, E) Pluripotency of the iPSCs was validated by immunofluorescence microscopy analysis (D) (Scale bar= 400 µm) and flow cytometry analysis (E) of the pluripotency markers OCT3/4, SSEA-4 and TRA-1-60. (F, G) iPSC clones successfully formed EBs (F) and spontaneously differentiated into the three germ layers (G). G) Expression of ectoderm (α-Internexin neurofilament (NF66)), mesoderm (α-SMA) and endoderm (α-FP)) was confirmed by immunofluorescence microscopy analysis.

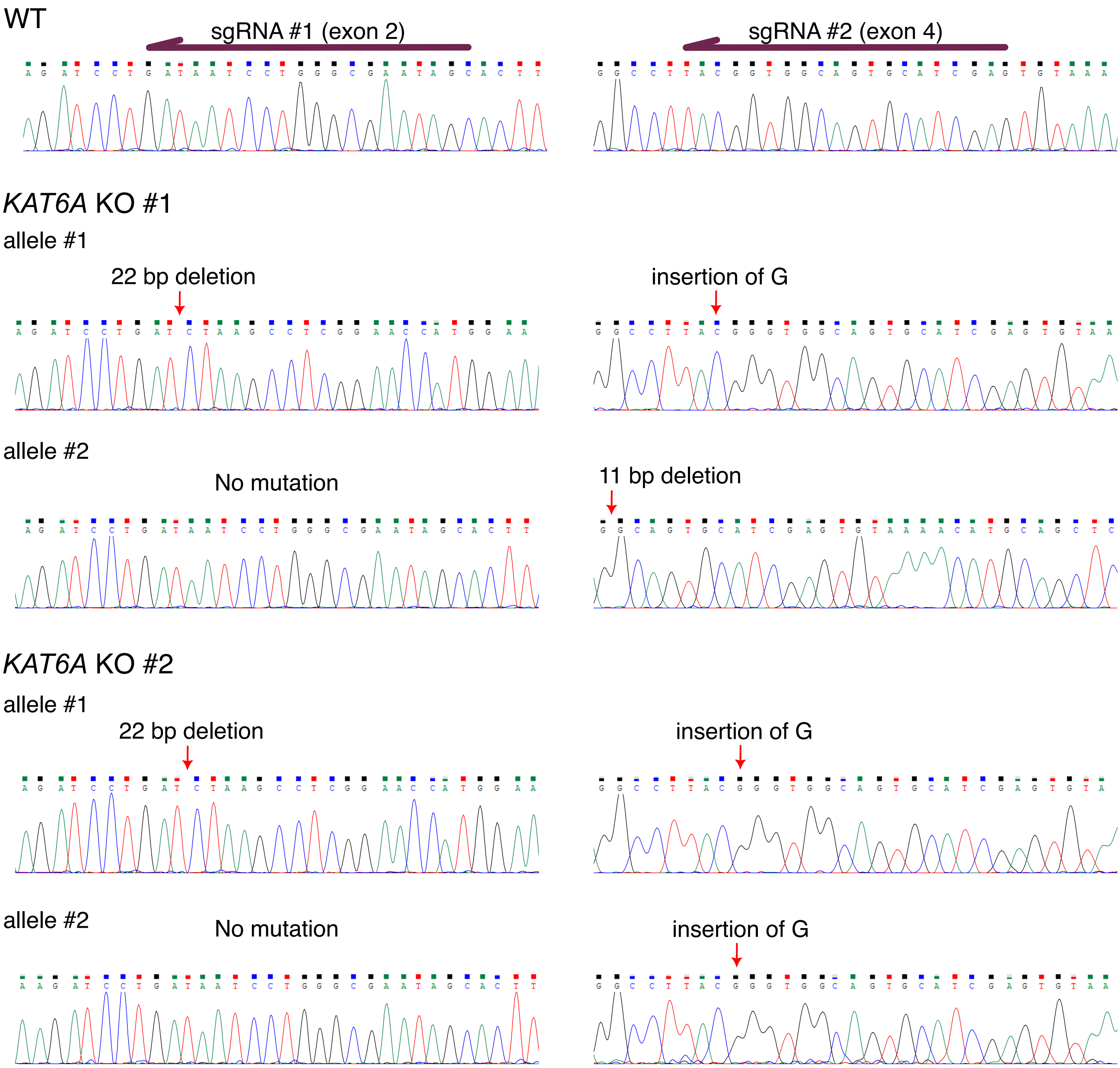

Figure S2. Validation of *KAT6A K*O in SH-SY5Y cells via Sanger DNA sequencing. Sanger sequencing results of WT control and edited cell lines (*KAT6A* KO clone #1/clone #2). Thirty-one bases of the chromatogram are shown. The sgRNA sequences targeting the *KAT6A* gene are indicated on the WT sequence.

Supplementary Tables S1-3. RNA sequencing results of dysregulated genes and pathways in NPCs (Table S1), cortical neurons at the 1st tp of differentiation (Table S2), and cortical neurons at the 2nd tp of differentiaion (Table S3).

Supplementary Table S4. Protein-coding genes that are dysregulated in *KAT6A*-mutant NPCs and cortical neurons, which are also associated with ASD according to the SFARI database.

Supplementary Table S6. A detailed description of all the quantified morphometric parameters .

Supplementary Table S5. List of primers for PCR, sequencing, and RT-PCR.

Primers for *KAT6A* sequencing	
KAT6A fragment amplification for iPSCs seq:	
KAT6A F	TTGCGAAAACGTGATGTGAAG
KAT6A R	GGGAGTCTTCTTCGTGGTCGT
KAT6A seq (iPSCs)	CAGAGGAGGAGCAGCAGGAA
KAT6A fragment amplification for SH-SY5Y clones seq:	
KAT6A F	TGGTAAAACTCGCAAACCCG
KAT6A R	GATCTCGACAGGAGCTGCATG
KAT6A seq (SH-SY5Y clones)	TGGTAAAACTCGCAAACCCG
Primers for RT-PCR	
KAT6A F	GCTGAACCAATCCCCATCTGT
KAT6A R	TCTCGACAGGAGCTGCATGTT
KAT6B F	GGATTTGGACGGTTTCTCATTG
KAT6B R	GCCTTGATGCTGATGTGCCT
NTRK2 F	TTGAAGCTTATGTGGGACTGAGA
NTRK2 R	TCAGACAAGTCAAGGTGACGGA
PSD95 F	AAACCAGAAGAGTACAGCCGATT0
PSD95 R	GCCTGCCACCACTCCTCAT
SYP F	GAAGGTGCTGCAATGGGTCTT
SYP R	GGTGCAGCCTGAAGGGGTA
ENO2 F	GGAGAACAGTGAAGCCTTGGA
ENO2 R	CAATCATCCTGGTCAAATGGGT
RSRC1 F	TGTTCAGCAGACATTCAGATCA
RSRC1 R	CAGTACTGGGTGGATCAGCA
*CRABP*2 F	CCCTGTAAGAGCCTGGTGAAAT
CRABP2 R	GCCGTCATGGTCAGGATCAGT
SHOX2 F	CCCGAGTGCAGGTTTGGTT
SHOX2 R	AAATGGCATCCTTAAAGCACCTA
EMC7 F	CAGACTGCCCTATCCTCTCCA
EMC7 R	TCAGGCAACTCATGGTTGGA
GAPDH F	ATGGGGAAGGTGAAGGTCG
GAPDH R	GGGGTCATTGATGGCAACAATA
RPLP0 F	GCACCATTGAAATCCTGAGTGA
RPLP0 R	ACATTGCGGACACCCTCCA
